# BCR-ABL1-driven exosome-miR130b-3p-mediated gap-junction Cx43 MSC intercellular communications imply therapies of leukemic subclonal evolution

**DOI:** 10.7150/thno.83178

**Published:** 2023-07-03

**Authors:** Chengyan Chai, Ke Sui, Jun Tang, Hao Yu, Chao Yang, Hongyang Zhang, Shengwen Calvin Li, Jiang F. Zhong, Zheng Wang, Xi Zhang

**Affiliations:** 1 Medical Center of Hematology, Second Affiliated Hospital, Army Medical University, Chongqing,400037, China.; 2 State Key Laboratory of Trauma, Burn and Combined Injury, Army Medical University, Chongqing, 400037, China.; 3 Jinfeng Laboratory, Chongqing, 401329, China.; 4 Neuro-Oncology and Stem Cell Research Laboratory, Center for Neuroscience Research, CHOC Children's Research Institute, Children's Hospital of Orange County (CHOC), 1201 La Veta Ave., Orange, CA 92868-3874, United States of America.; 5 Department of Neurology, University of California-Irvine School of Medicine, 200 S. Manchester Ave. Ste. 206, Orange, CA 92868, United States of America.; 6 Department of Basic Sciences, Loma Linda University, Loma Linda, California, 92354, United States of America.

**Keywords:** acute lymphoblastic leukemia, B progenitor acute lymphoblastic leukemia, leukemic microenvironment, MSCs, BCR-ABL1, miR-130a and miR-130b, gap junctions, Cx43, stemness, chemotherapy resistance

## Abstract

**Rationale:** In the bone marrow microenvironment (BMME), mesenchymal stem/stromal cells (MSCs) control the self-renewal of both healthy and cancerous hematopoietic stem/progenitor cells (HSPCs). We previously showed that *in vivo* leukemia-derived MSCs change neighbor MSCs into leukemia-permissive states and boost leukemia cell proliferation, survival, and chemotherapy resistance. But the mechanisms behind how the state changes are still not fully understood.

**Methods:** Here, we took a reverse engineering approach to determine BCR-ABL1+ leukemia cells activated transcriptional factor C/EBPβ, resulting in miR130a/b-3p production. Then, we back-tracked from clinical specimen transcriptome sequencing to cell co-culture, molecular and cellular assays, flow cytometry, single-cell transcriptome, and transcriptional regulation to determine the molecular mechanisms of BCR-ABL1-driven exosome-miR130b-3p-mediated gap-junction Cx43 MSC intercellular communications.

**Results:** BCR-ABL1-driven exosome-miR130a/b-3p mediated gap-junction Cx43 (a.k.a., GJA1) BMSC intercellular communications for subclonal evolution in leukemic microenvironment by targeting BMSCs-expressed HLAs, thereby potentially maintaining BMSCs with self-renewal properties and reduced BMSC immunogenicity. The Cx43^low^ and miR-130a/b^high^ subclonal MSCs subsets of differentiation state could be reversed to Cx43^high^ and miR-130a/b^low^ subclones of the higher stemness state in Cx43-overexpressed subclonal MSCs. Both miR-130a and miR-130b might only inhibit Cx43 translation or degrade Cx43 proteins and did not affect Cx43 mRNA stability. The subclonal evolution was further confirmed by single-cell transcriptome profiling of MSCs, which suggested that Cx43 regulated their stemness and played normal roles in immunomodulation antigen processing. Thus, upregulated miR-130a/b promoted osteogenesis and adipogenesis from BMSCs, thereby decreasing cancer progression. Our clinical data validated that the expression of many genes in human major histocompatibility was negatively associated with the stemness of MSCs, and several immune checkpoint proteins contributing to immune escape in tumors were overexpressed after either miR-130a or miR-130b overexpression, such as CD274, LAG3, PDCD1, and TNFRSF4. Not only did immune response-related cytokine-cytokine receptor interactions and PI3K-AKT pathways, including EGR3, TNFRSF1B, but also NDRG2 leukemic-associated inflammatory factors, such as IFNB1, CXCL1, CXCL10, and CCL7 manifest upon miR-130a/b overexpression. Either BCR siRNAs or ABL1 siRNAs assay showed significantly decreased miR-130a and miR-130b expression, and chromatin immunoprecipitation sequencing confirmed that the regulation of miR-130a and miR-130b expression is BCR-ABL1-dependent. BCR-ABL1 induces miR-130a/b expression through the upregulation of transcriptional factor C/EBPβ. C/EBPβ could bind directly to the promoter region of miR-130b-3p, not miR-130a-3p. BCR-ABL1-driven exosome-miR130a-3p could interact with Cx43, and further impact GJIC in TME.

**Conclusion:** Our findings shed light on how leukemia BCR-ABL1-driven exosome-miR130b-3p could interact with gap-junction Cx43, and further impact GJIC in TME, implications for leukemic therapies of subclonal evolution.

## Introduction

Translocation between chromosomes 9 and 22 causes the production of the oncogenic fusion protein BCR-ABL1, constitutively active and responsible mainly for the development of chronic myeloid leukemia (CML) and a small proportion of B progenitor acute lymphoblastic leukemia (B-ALLs). These fusion-derived mutations result in constitutively active tyrosine kinases, eventually contributing to leukemogenesis 1. The development of BCR-ABL1 targeted therapies, such as multiple tyrosine kinase inhibitors (TKI), has dramatically changed the treatment landscape for CML 2. E.g., Tyrosine kinase inhibitors are the gold standard treatment for CML. Despite the advancements in TKI therapies, however, the effectiveness is always short-term in an advanced phase of CML or acute lymphoblastic leukemia (ALL) that expresses BCR-ABL1 due to the subclonal evolution of tumor heterogeneity developing drug resistance 3, including the subclonal acquisition of a BCR::ABL1 fusion in CML 4 and of that KMT2A rearrangement, DEK-NUP214 fusion, and NPM1 mutation are associated with the upregulation of HOX genes plus concurrent mutations of ASXL1 and RUNX1 in AML^5^, as well as point mutations (T315I mutations) in the BCR-ABL1 fusion gene in CML^6^. The resistance to TKI is driven by either BCR-ABL1-dependent 7 or independent 8 mechanisms. On the BCR-ABL1-dependent, the activating mutations in the kinase domain of BCR-ABL1 can progressively accumulate the resistance to TKI by compromising *imatinib* binding to the steric clash, thereby directing contact elimination or changing ABL1-kinase conformation 9. On the other hand, for BCR-ABL1-independent by reactivating downstream effectors of signaling transduction of BCR-ABL1 10 or affecting cancer microenvironmental factors 11, TKI resistance can also occur independently of BCR-ABL1 inhibition. For example, high STAT5 expression in CML indicates resistance to TKI 12. The paradoxical activation of the MEK/ERK pathway driven by RAF phosphorylation also contributes to TKI resistance 13. Unfortunately, these studies still fail to decipher leukemia recurrence fully.

The bone marrow microenvironment (BMME) controls normal and malignant hematopoietic cell self-renewal, as well as Piezo1-mediated mechanosensation for vascular niche regeneration after irradiation injury 14. The primary component of the BMME is bone marrow mesenchymal stromal cells (BMSCs), which can differentiate into osteoblasts, adipocytes, and chondroblasts. Recent evidence suggests that leukemia cells remodel the BM stromal components via the extracellular matrix to improve drug resistance, proliferation, and stress-induced quiescence 15. For instance, due to the extracellular matrix-vascular cell adhesion molecule-1 (VCAM-1) overexpression, BMSCs protect leukemia cells from cytotoxicity caused by cytarabine and etoposide treatment 16. Furthermore, as a potential source of tumor cancer-associated fibroblasts (CAFs), MSCs exert similar characteristics of pro-tumorigenic and immunomodulation properties 17. A number of chemokines and cytokines mainly secreted by MSCs, including placental growth factors (PGF), macrophage inflammatory protein 1 alpha/beta (MIP-1a/b), IL-1a/b, IL-8 and TNF-α 18, are in more significant quantities in the leukemic microenvironment (LME), which can support the growth of leukemia cells. Besides, LME also promotes tumor malignancy by the disturbance of immune cells. The plasticity of LME remodeled by AML cells prevents the activation and proliferation of T cells through the inhibition of NF-kB, c-Myc, and pRb pathways 19. Tregs formed in LME can be inducted and survived by elevated enzymes involved in metabolisms, such as Indoleamine 2,3-Dioxygenase 1 (IDO1) and Arginase II 20. Thus, identifying signaling pathways within intercellular communication networks of LME is critical for the potential development of novel therapeutics. Gap junctions (GJs) mediate intercellular communication between hematopoietic cells and BMSCs 21. The impairment of Cx43 in the BM stromal components functionally directly induces normal hematopoietic failure and B lymphopoietic defects 22. Changes in the expression and function of Cx43 in immune cells resulted in alternative inflammatory responses and immune dysfunction 23. Consistent with other reports 24, we previously showed that the decreased Cx43 expression is associated with the disturbed GJIC function of BMSCs in LME 25, 26. Conversely, overexpression of Cx43 in BMSCs enhances AML apoptosis and reduces leukemia cells' anti-drug resistance 27. However, the mechanisms associated with the disturbance of Gap junctional intercellular communications (GJICs) Cx43 are still unknown in LME.

In solid tumors, many studies showed that cancer cells featured a lack of gap junction proteins or reduced channel activities, resulting in poor GJs coupling, revealing that gap junction proteins may serve as tumor suppressors 28. However, the effects of heterotypic gap junctions on tumor cells and immune cells lead to either the pro-tumor or anti-tumor responses, suggesting its complicated roles in cell communications within the tumor microenvironment (TME) 29. Previous studies showed that GJs activated STING signaling pathways and elevated the levels of IFN-a and TNF cytokines, promoting tumor cell growth and chemoresistance 30. Cx43-mediated GJICs play a vital role in the suppressive capacity of Treg cells 31. On the other hand, in the melanoma microenvironment, GJs such as Cx43 supported the preprocessing of tumor-associated antigens, improving T-cell activation and anti-tumor immunity 32. In addition, Cx43 is required for immune cell development, such as T and B lymphopoiesis 33. Cx43-mediated GJICs could also be a positive regulator of B cell motility, CXCL12-induced cell migration, and trans-endothelial migration. Therefore, the roles of Cx43-mediated GJICs in immune responses of TME are still elusive.

Extracellular vehicles (EVs) function in intercellular signaling transduction in TME, and EVs influence TME to promote cancer metastasis and progression 34. On matched patient-derived organotypic tumor spheroids (PDOTS) and matched patient-derived organoids (PDOs) that mimic TME, targeting innate immune kinase TANK-binding kinase 1 (TBK1) (an immune evasion gene) enhances response to PD-1 blockade by lowering the cytotoxicity threshold to effector cytokines (TNFα/IFNγ) thereby overcoming resistance to cancer immunotherapy 35. A link between leukemia cell-secreted exosomes and their educated stromal cells has been well established to illustrate the molecular identities and characteristics of LME. For instance, exosomes released from CML stimulate BMSCs to produce IL-8 and support leukemic growth 36. Similarly, acute myeloid leukemia (AML)-derived exosomes also transformed the bone marrow niches into a leukemia-permissive microenvironment, contributing to leukemia growth and disruptions of normal hematopoiesis 37. Notably, immune suppression and tumor progression can be enhanced by CML-secreted exosomes 38. Furthermore, miRNAs in exosomes derived from leukemic TME (LME) are critical regulators of leukemogenesis and relapse. E.g., miRNA-126 released from CML exosomes modulated CML progression by directly targeting the 3'-UTR of CXCL12 and VCAM1 mRNA 39. Overexpressed miR-155 promoted AML leukemogenesis targeting C/EBPA 40, and exosomal miR-155 increased AML cells' capacity for drug resistance against TKIs 41. In AML, miR-130a has been considered an adverse outcome predictor and a therapeutic target 42. Overexpressed miR-130b contributes to leukemogenesis in patients with acute leukemia and acute promyelocytic leukemia (APL) 46. However, the functional roles of miR-130a/b in leukemogenesis and relapse are still unknown.

Reflecting on the gaps of knowledge in the above literature, we started with initiated finding that Cx43-modified bone marrow stromal cells reverse the imatinib resistance of K562 cells via Ca 2+ -dependent gap junction 44. Following up, here we identified exosomes as a mediator for LME's intercellular communication. Specifically, we first found that exosome miRNAs are associated with BCR-ABL1 in CML and B-ALL patients. Second, we determined that leukemia cell-derived exosomes target BMSCs and impaired GJICs of healthy BMSCs. Third, we identified that Cx43 is a direct target of miR-130 a/b in BMSCs cells regulated GJICs. Fourth, we mapped out that miR-130a/b promotes the immunosuppressive property of BMSCs through BMSC stemness and differentiation regulation at the BCR-ABL1-mediated C/EBPβ transcriptional level. Lastly, BCR-ABL1 induces miR-130a/b expression through the upregulation of transcriptional factor C/EBPβ and C/EBPβ could bind directly to the promoter region of miR-130b-3p, not miR-130a-3p. Thus, we established a link between exosome regulating GJICs and LME reprogramming, pointing to the roles of BCR-ABL1-driven miRNAs in pro-leukemic education of BMSCs, and implications for subclonal evolution of therapy resistance.

## Results

We applied a comprehensive and systematic approach using molecular cell biology and biochemical techniques plus cancer genome-scale bioinformatics to demonstrate how miRNAs driven by BCR-ABL1 were involved in the education of pro-leukemic BMSCs, which potentially leads to subclonal evolution of therapeutic resistance, a mechanism by which cancer progression and posttreatment recurrence by subclonal switchboard signaling that shifts the dormant subclones to dominating subclones as previously described 3.

### The expression of miR-130a/b was associated with BCR-ABL1 in CML and B-ALL patients

**Figure 1:**
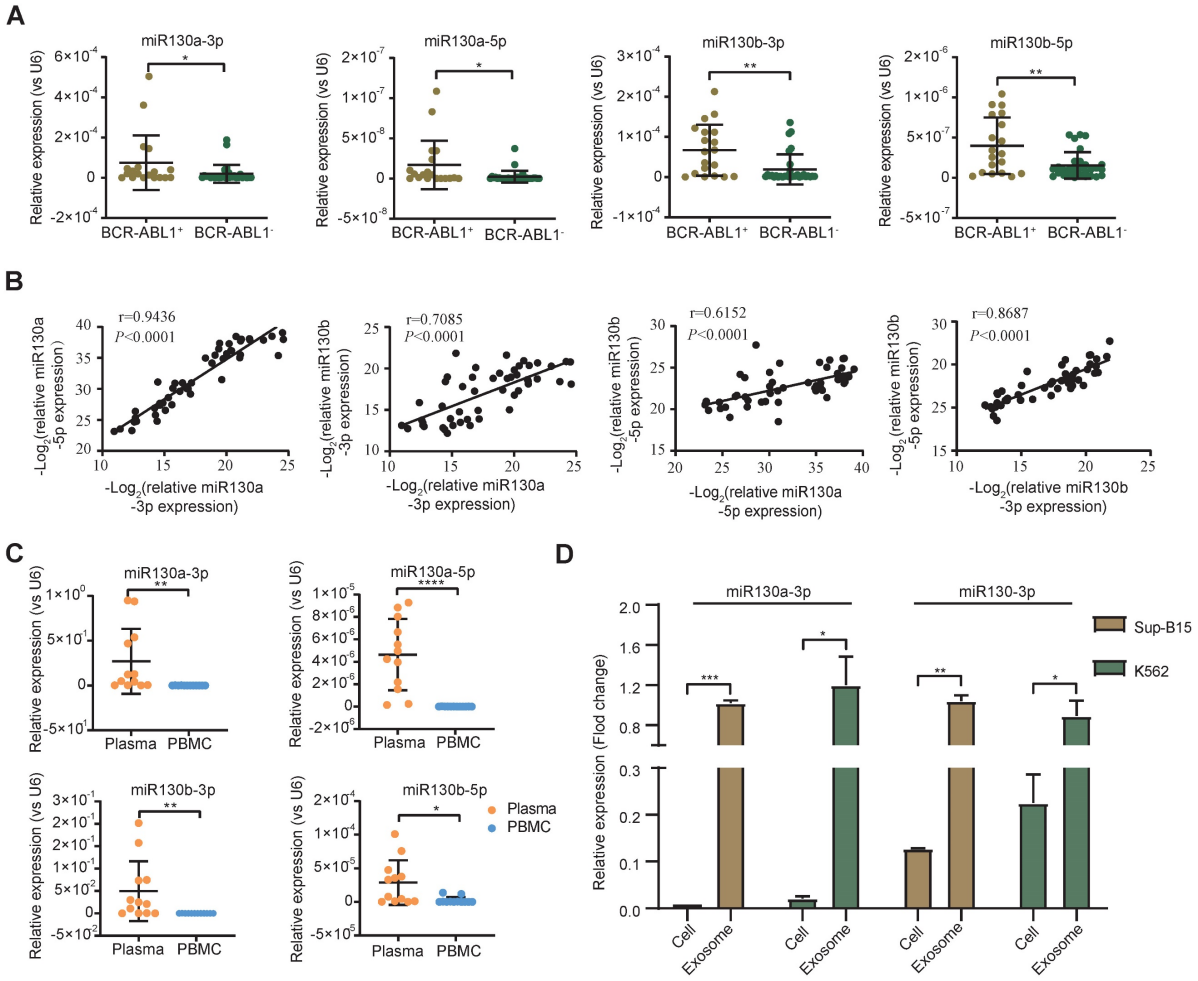
** miR-130a/b expression in BCR-ABL1 positive B-ALL patients. a** RT-qPCR analysis of miR130a-3p, miR130a-5p, miR130b-3p and miR130b-5p in BM aspirates from B-ALL leukemia. BCR-ABL1 positive (n=19) or negative B-ALL (n=30). **b** Pairwise miRNA correlation analysis for these miRNA expressions from total B-ALL samples (n=49). **c** The relative expression of miRNAs in paired plasma and PBMC samples from BCR-ABL1 positive samples (n=12). **d** The relative expression of miR130a/b-3p in cells versus exosome from Sup-b15 and K562 cell lines. The expression level of miRNAs was normalized against U6. Fold changes were normalized by expression levels in exosomes. The miRNA expression level was normalized against U6. Data are shown as mean ± SD representing three biological replicates. *P < 0.05, **P < 0.01, ***P < 0.001, by student's paired t-test.

Overexpression of the miRNA-130 family was significantly associated with poor survival in many solid tumors 45. In AML and APL, overexpressed miR-130a or miR-130b was associated with adverse outcomes, respectively 42, 46. However, the expression of miR-130a/b in BCR-ABL1^+^ CML and B-ALL patients was still unclear. To evaluate the alternative miR-130a/b expression in the leukemia initiation stage, we measured the relative expression of miRNA 5p/3p strands of miR-130a/b in diagnostic BM aspirates from primary BCR-ABL1^+^ B-ALL and BCR-ABL1^-^ B-ALL samples, respectively. We found that the expression of miR-130a/b was significantly higher in BCR-ABL1^+^ B-ALL patients than in BCR-ABL1^-^ B-ALL patients (Figure 1A). Similarly, miR-130a/b was also increased dramatically in BCR-ABL1^+^ CML, compared to healthy individuals (Figure S1A). A strong correlation between 5p and 3p strands of the same miRNAs suggested their miRNA precursors were also overexpressed. Furthermore, the high correlation between miR-130a and miR-130b pointed out the high potential of functional collaboration and expression regulation in the miR-130 family** (**Figure 1B). Double-strands pre-miRNAs were processed into independent two-strand mature miRNAs via several key steps. In most cases, only one strand of mature miRNA is bound to the RNA-induced silencing complex (RISE) and functions as a post-transcriptional gene regulator, and the other mature miRNA is degraded 47. Similar to the above findings, we found that the 3p strand of both miR-130a and miR-130b was ~100 times higher than their respective 5p stand of miRNAs (Figure S1B, C), based on the RT-qPCR analysis of miR130a-3p, miR130a-5p, miR130b-3p, miR130b-5p in diagnostic BM aspirates from patients with BCR-ABL1 positive samples or BCR-ABL1 negative patients. This data indicated that the miRNA-130 family was processed via the canonical 3p-arm preference style in both B-ALL and CML.

To further explore the origination of the overexpressed miR-130a and miR-130b in BM aspirates, their relative expression between plasma and cells of BM aspirates was analyzed. The data indicated that the expression of miR-130a/b was much higher in plasma than in BM aspirate cells in both CML and ALL patients (Figure 1C). This result revealed that the overexpressed miR-130a/b was mainly derived from plasma in the leukemic BM microenvironment of patients with CML and ALL. We wanted to determine how the overexpressed miR-130a/b affected the components of the leukemic BM microenvironment.

Exosomes as small vesicles are wildly detected in blood plasma, urine, and other biological fluids. Several studies found that some miRNAs can be preferentially enriched into exosomes through sorting mechanisms 48. In K562 (the first human immortalized myelogenous leukemia cell line) and SUP-B15 (B lymphoblast cell line) - both cell lines are BCR-ABL1^+^, we also found that the enrichments of the respective miRNAs in exosomes were significantly higher than those in total cell lysates (Figure 1D). Our data are consistent with the exosomal miRNAs are the enriched source of circulating miRNAs, perhaps because exosome-captured miRNAs are shielded from cell-free RNase so that they are barriered from RNase and reduced miRNA degradation 49. Exosomes might contribute to acellular communication, which results in the transfer of molecules between cells when multivesicular endosomes fuse with the cell surface in the extracellular environment 50. Exosomes were postulated to regulate immune responses, which might be viable in cancer immunotherapy. Uncertainty exists regarding the mechanisms underlying exosome secretion and contacts with target cells, which prompted us to search for their target cells within the leukemic BM microenvironment (BME, LME).

### Leukemia cells-derived exosomes target BMSCs and impaired GJICs of healthy BMSCs

**Figure 2:**
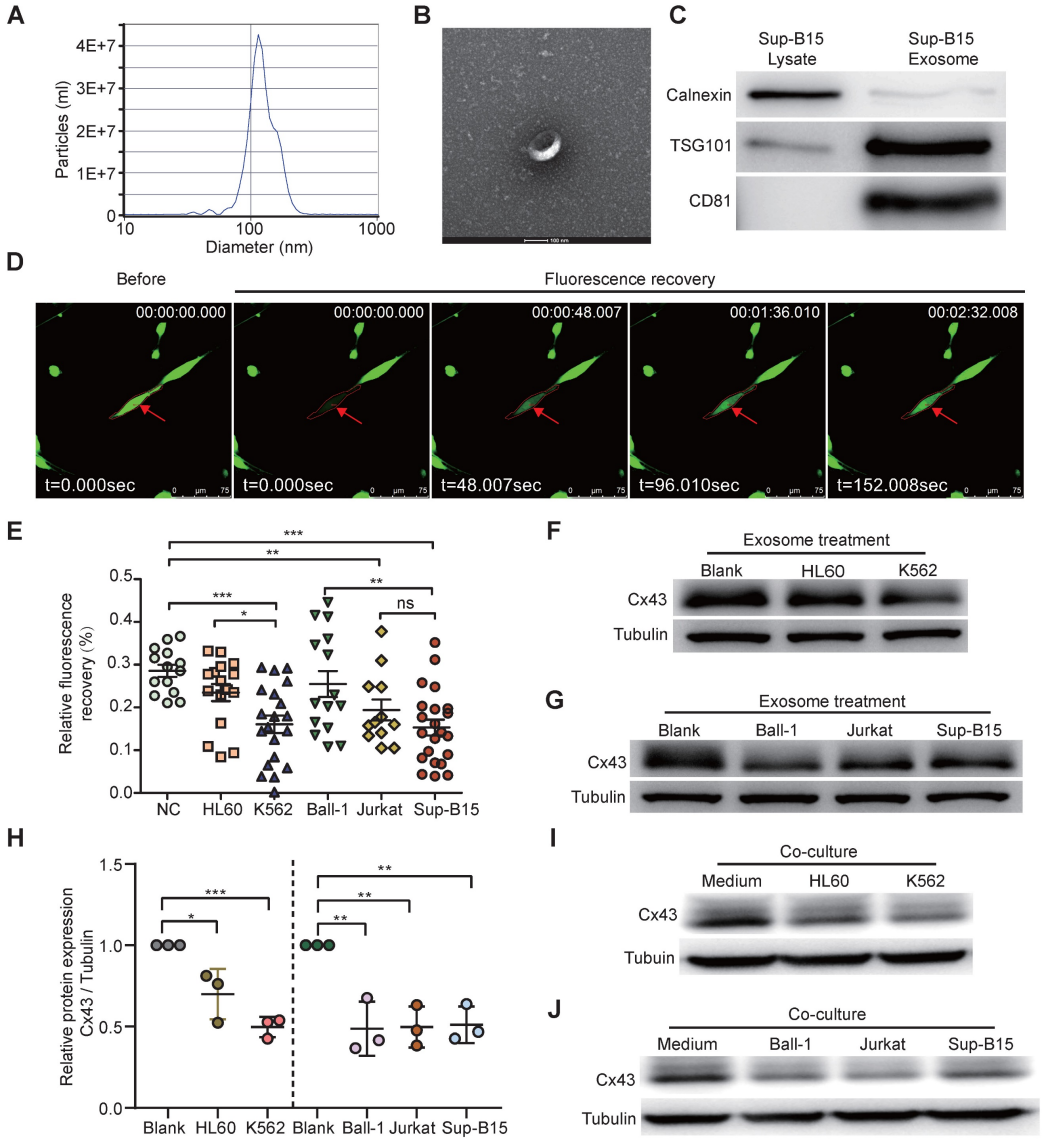
** Exosomes derived from leukemia cells impaired GJIC of BMSCs. a** Nanoparticle tracking analysis showed the size distribution of exosomes derived from Sup-B15 cells. **b** Exosomes were imaged by Electron microscopy, revealing the typical morphology and size. Scale bar=200 nm. **c** Western blotting analysis of TSG101, CD81 and Calnexin in exosomes from Sup-B15 cells. **d** Representative images of gap junction communication of BMSCs were assessed by measuring FRAP. Photobleached BMSC was marked with red arrows. **e** Statistical analysis of FRAP results for BMSCs treated by exosomes derived from HL60, K562, Ball-1, Jurkat, and Sup-B15, respectively.** f, g** showed representative images of western blotting. The expression levels of Cx43 in BMSCs treated by exosomes derived from multiple leukemia cell lines, respectively. **h** Fold changes were normalized by the blank group. **I, j** showed representative images of western blotting after co-culture treatment. The relative levels of Cx43 in BMSCs co-culturing with multiple leukemia cell lines, respectively. Mean ± SD representing more than three biological replicates. *P < 0.05, **P < 0.01, ***P < 0.001, by student's unpaired t-test.

Many studies showed BMSC aberrations within BME in several types of human hematologic malignancies, such as AML and MDS 51, 52. BMSC heterogeneity has been widely reported in different passages and donors; however, the causative factors have not been fully characterized. We hypothesized that leukemic cell-derived exosomes target BMSCs. To better investigate how leukemia-derived exosomes induce BMSC changes, BMSCs derived from healthy donors were isolated and identified (Supplementary Figure 2A, B) according to previous methods 53. Exosomes from the cell culture medium of either BCR-ABL1^+^ (Sup-B15 and K562) cell lines or BCR-ABL1^-^ (Ball-1, HL60, and Jurkat) leukemia cell lines were isolated, respectively, using ultracentrifugation. To characterize the quality of isolated exosomal vesicles, size, morphology, and classical surface markers of exosomes were first examined by transmission electron microscopy and Western blotting (Figure 2A-C). The data showed that the size distribution of the isolated exosomes was 40-150 nm in diameter (Figure 2A, B), and the exosomes contained the well-established exosome positive markers TSG101, CD81, and negative marker calnexin indicated their high quality (Figure 2C). Furthermore, fluorescently labeled exosomes derived from leukemic cells K562 or Sup-B15 cells could be within BMSCs after 72h of co-culture (Figure S3A, B).

To study the alternative functionality of GJICs by leukemic exosomes, we measured the GJIC capacity of BMSCs by fluorescence recovery after photobleaching (FRAP) using confocal microscopy living image after leukemic exosome treatment (Supplementary Figure 3C). After 48 hs of exosome treatment, FRAP analysis showed that each leukemia cell line-derived exosome could significantly reduce the fluorescence recovery rate of bleached BMSCs, compared to the untreated control group (Figure 2D, E). Furthermore, exosomes from BCR-ABL1^+^ leukemia cell lines obviously delayed the fluorescence recovery rate compared to those from BCR-ABL1^-^ leukemia cell lines (Figure 2E). In addition, the protein level of Cx43 was significantly downregulated after exosome treatment compared to the untreated control group (Figure 2F-H and Figure S4A). To exclude the specifically phenotypic possibility of exosome isolated by ultracentrifugation, we reproducibly performed the same experiments by exosomes purified using membrane affinity spin columns (exoEasy Maxi Kit) and obtained similar results (Supplementary Figure 4B). Consistently, the expression of Cx43 in BSMCs was also obviously decreased after 72h of co-culture with leukemic cells (Figure 2I-J and Figure S4C). These data suggested that leukemia-cell-derived exosomes disturbed the capacity of GJs and reduced Cx43, especially for BCR-ABL1^+^ leukemia-cell-derived exosomes. Next, we wanted to search for Cx43 upstream activators and downstream effectors.

### Cx43 is a direct target of miR-130 a/b in BMSCs cells

**Figure 3:**
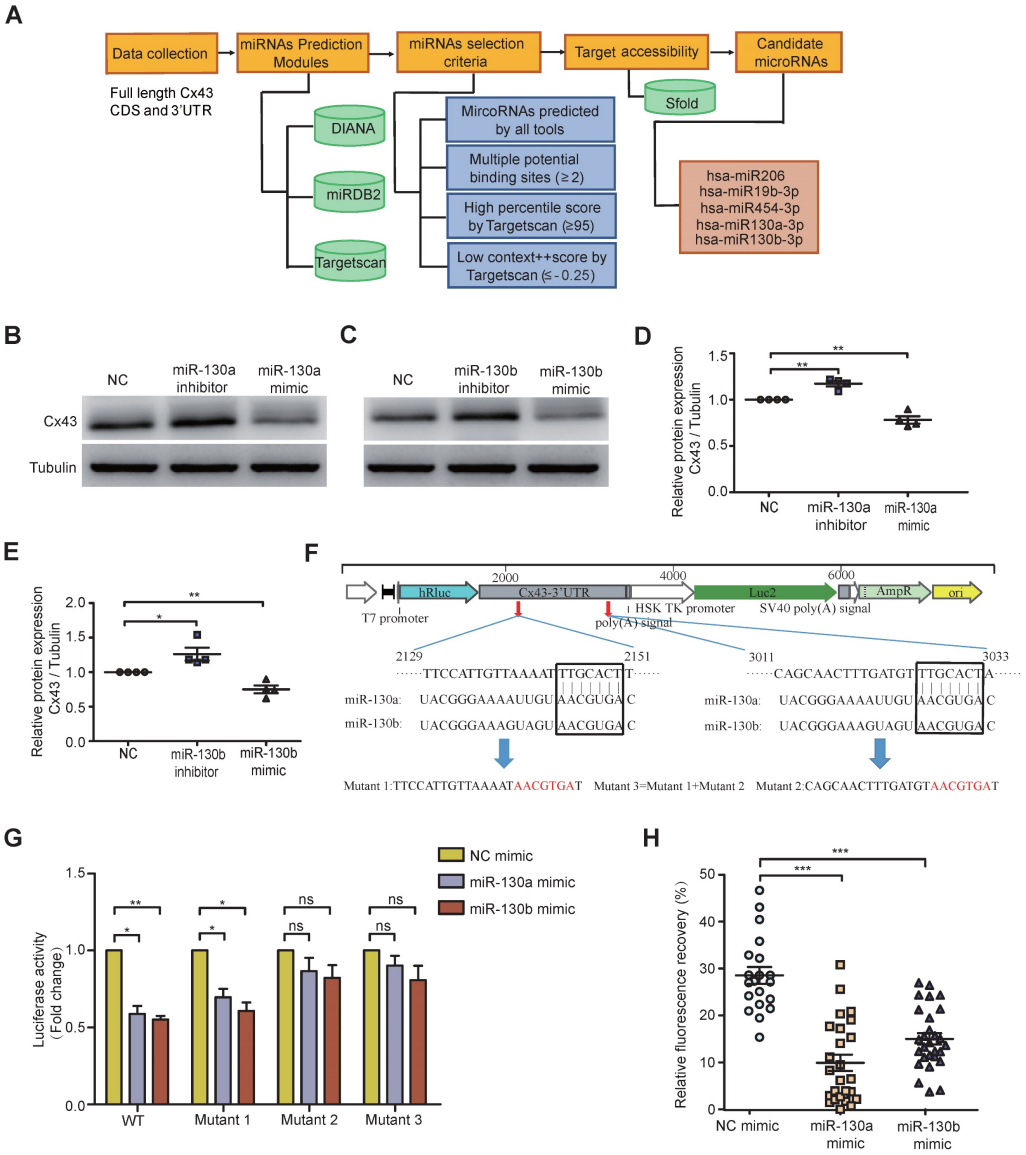
** Both miR-130a and miR-130b target Cx43 and impair the GJIC of BMSCs. a** Bioinformatics pipeline prediction showing miR-130a/b targeting mRNA of Cx43. **b-e** Western blotting was applied to measure the relative protein levels of Cx43 in BMSCs after transfection with indicated or miR-130b-3p mimics or inhibitors for 48 hs (**b** and **c**). The results were quantified and normalized to NC groups and TUBULIN (**d** and **e**). mean ± SD representing 4 different donors for biological replicates. **f** The luciferase reporter vectors were constructed. Cx43-3' UTR WT or mutant sequences were inserted into psiCHECK2 plasmids, respectively. **g** Luciferase activity assay was performed and normalized to the luciferase activity of Cx43-3' UTR WT. **h** FRAP assays showed that miR-130a-3p and miR-130b-3p had an impact on the gap junction activity of BMSCs, respectively. Each dot represents one observed BMSC. *P < 0.05, **P < 0.01, ***P < 0.001, by student's unpaired t-test.

Exosomal miRNAs were widely investigated from donor cancer cells to recipient cells and miRNA-mediated gene silencing. To computationally predict potential miRNAs targeting Cx43, several popular databases such as Targetscan, miRDB2, and DIANA were used. Based on full-length Cx43 CDS and 3'-UTR, selection criteria, including seed match, binding sites, target accessibility, high percentile score, and low context score, were performed to choose the most highly potential miRNAs (Figure 3A). Only 5 miRNA candidates met the selection criteria, including miR-130a and miR-130b, which were significantly overexpressed in BCR-ABL1^+^ B-ALL and CML (Figure 1A and Figure S1A). Their mimics or inhibitors were overexpressed to validate whether miR-130a or miR-130b could directly regulate Cx43 expression; their mimics or inhibitors were overexpressed in BMSCs. Real-time PCR or Western blotting was performed 48 or 72 hs after transfection. The results showed that the mRNA level of Cx43 was not significantly changed (Figure S5A), while its protein level was reduced considerably after their mimic's transfection (Figure 3B-E).

Furthermore, the mRNA level of Cx43 was not negatively correlated with the expression of either miR-130a or miR-130b in leukemia samples (Figure S5B, C). All these results suggested that miR-130a and miR-130b might only inhibit Cx43 translation or degrade Cx43 proteins, and they did not affect Cx43 mRNA stability. Conversely, both of their inhibitors significantly increased their protein levels (Figure 3B-E and Figure S6A). The computational prediction suggested two putative binding sites for both miR-130a and miR-130b (Figure 3F). A dual-luciferase reporter psiCheck2 vector was constructed by inserting the wild-type or mutant 3'-UTR of the human Cx43 gene into the 3' end of the *renilla* luciferase gene. At 24-30 hs after transfection with the reporter vector and either miR-130a, miR-130b, or negative control (NC) oligonucleotides into 293T cells, luciferase activity was measured. These results showed that the luciferase activity was significantly repressed by miR-130a and miR-130b. Only mutations in binding site 2 and both sites could efficiently reduce the suppression by both miR-130a and miR-130b (Figure 3G).

Moreover, FRAP analysis showed that overexpressed miR-130a or miR-130b could also greatly disturb the capacity of GJICs in BMSCs (Figure 3H and Figure S6B), like exosome treatment results (Figure 2E). Through transcriptome sequencing of normal BMSCs, Cx43 was the highest abundant connexin protein among the Connexin family, consisting of 21 members as checked in humans. In contrast, other members were relatively low relative (Figure S5D). In this study, we found that the expression of miR-130a/b was associated with BCR-ABL1 in CML and B-ALL patients. To further find the potential targets of miR-130a/b-3p, we performed the bioinformatics analysis to predict the target genes of miR-130a/b-3p using TargetScan. We found that the only downstream protein in the family of gap junctions was Cx43/GJA1. Overexpression of miR-130a or miR-130b had no apparent effects on the mRNA of other connexins (Figure S5E); however, GJA1, the target gene, seemed to exhibit the opposite trend in that its expression was increased after miR-130a/b overexpression, especially miR-130b overexpression (Figure S5E). Taken together, these results confirmed that the overexpression of miR-130a/b can directly reduce Cx43 and damage the GJs of BMSCs. We followed up with a question about the impact of the damaged GJs on the fate of BMSCs.

### miR-130a/b inhibits osteogenesis and promotes adipogenesis from BMSCs

**Figure 4:**
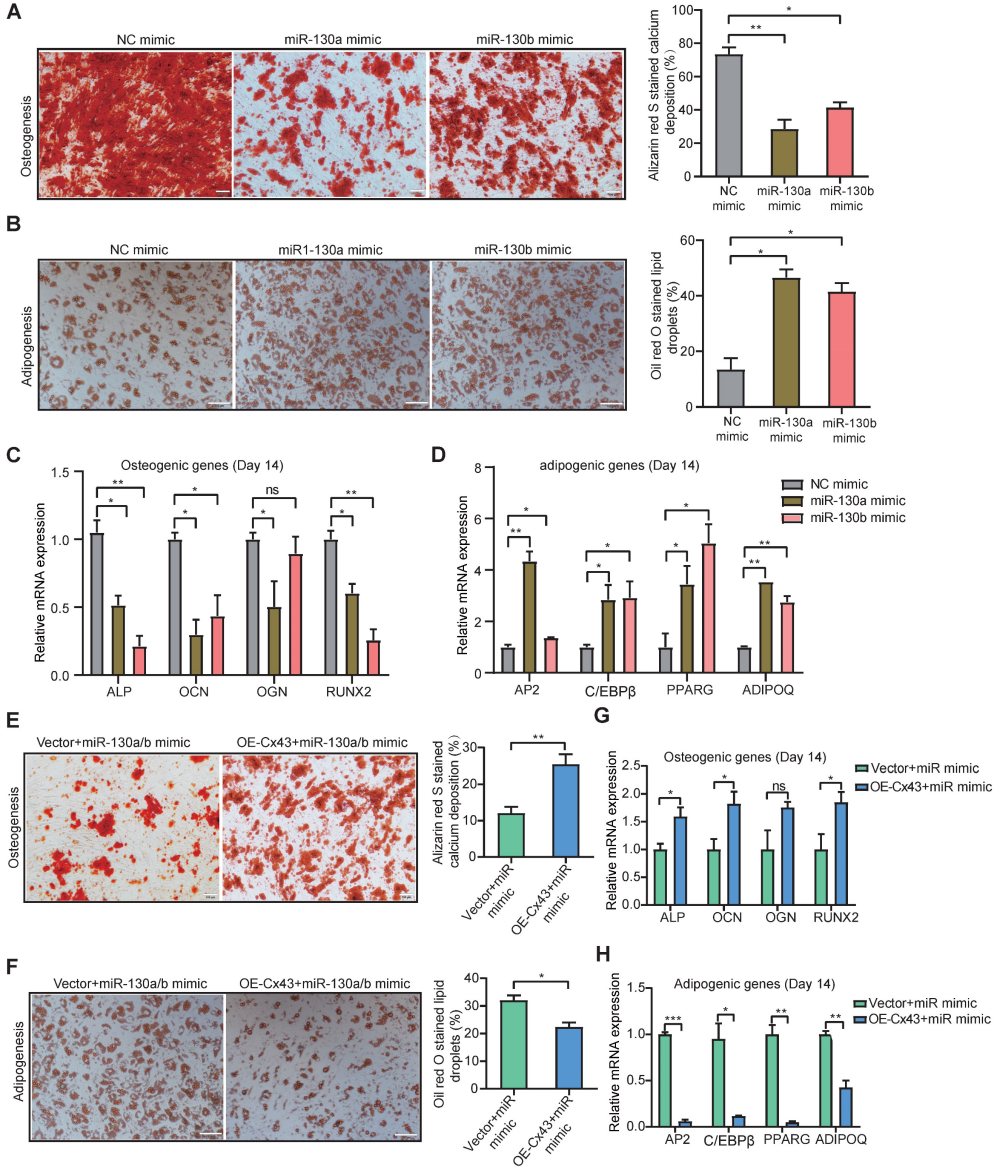
** Both miR-130a and miR-130b inhibits osteogenic differentiation and promotes adipogenic differentiation of BMSCs. a, b** Representative images of Alizarin Red S (**a**) or Oil Red O (**b**). Staining of mock and miRNA overexpressing BMSCs, respectively. BMSCs were transduced with the lentiviral vectors pLV-EGFP-miR-130a/b or pLV-EGFP-mock vectors, respectively. The positive BMSCs were purified by puromycin screening. After purification, BMSCs were induced for 14 or 21 days in an osteogenic or adipogenic medium, respectively. Scale bars=100um. The positive stainings were quantitively analyzed in 8 random areas in each biological replicate using image pro plus 6.0. All experiments were replicated more than 3 times independently. **c, d** Real-time PCR analysis of osteogenic (**c**) and adipogenic (**d**) expression levels after osteogenic or adipogenic differentiation of BMSCs for 14 or 21 days, respectively. **e, f** BMSCs were co-infected with Cx43 overexpressing or control lentivirus with the mixed pools of miR-130a/b lentivirus in osteogenic or adipogenic medium for 14 days or 21 days, respectively. Representative images of Alizarin Red S (**e**) or Oil Red O (**f**) staining were shown, respectively. Scale bars=100 um. The positive stainings were quantitively analyzed in 8 random areas in each biological replicate using image pro plus 6.0. All experiments were replicated more than 3 times independently. **g, h** The relative expression levels of osteogenic (**g**) or adipogenic (**h**) genes were measured by Realtime PCR at the indicated time points, respectively. The relative mRNA levels of these genes were normalized by NC group and internal GAPDH expression, and mean ± SD represents 3 independent replicates in all the Real-time PCR experiments. *P < 0.05, **P < 0.01, ***P < 0.001, by student's unpaired t-test.

Previous reports showed that osteoblast reduction in the leukemic microenvironment (LME) results in the disturbance of bone formation and bone loss, thereby altering normal hematopoiesis 54. Gonadal adipose tissues were identified as a reservoir for leukemia stem cells (LSCs) to enhance fatty acid oxidation metabolism in LSCs and evade chemotherapy 55. To determine whether miR-130a/b can affect the fate of BMSCs, osteogenic and adipogenic differentiation capacities were investigated. The osteogenic and adipogenic differentiation assays showed both miR-130a and miR-130b significantly reduced the osteogenic differentiation potential of BMSCs *in vitro* (Figure 4A), while substantially increasing the adipogenic differentiation capacity of BMSCs *in vitro* (Figure 4B). Both adipogenic and osteogenic differentiation was verified by using the classically known biomarker genes related to osteogenesis and adipogenesis. Real-time PCR analysis showed that significantly lower osteogenic differentiation-associated genes, such as alkaline phosphatase (ALP), osteocalcin (OCN), osteoglycin (OGN), and runt-related transcription factor 2 (RNUX2) (Figure 4C), while the expression of adipogenic differentiation-associated genes, such as CCAAT enhancer binding protein beta (C/EBPβ), peroxisome proliferator-activated receptor gamma (PPARG), adipocyte protein 2 (AP2), adiponectin (ADIPOQ) was significantly increased *in vitro*, respectively (Figure 4D). To verify further whether miR-130a/b alters the BMSC differentiation by targeting Cx43, the overexpression of Cx43 was performed in miR-130a or miR-130b overexpressing BMSCs. The data showed that overexpressed Cx43 could significantly inhibit miR-130a or miR-130b, promoting adipogenesis and restoring the osteogenic differentiation capacity of BMSCs (Figure 4E, F). Realtime PCR analysis also validated the biomarker genes of osteogenic or adipogenic differentiation under two conditional mediums (Figure 4G, H). These results demonstrated that miR-130a/b could shift the preferential differentiation of BMSCs toward adipocytes than to osteoblasts. Which subclone of BMSC takes the tipping point of such a shifted preferential differentiation of BMSCs toward adipocytes as mediated by miR-130a or miR-130b-regulated Cx43? Thus, next, we wanted to characterize the heterogeneous Cx43 expression associated with the stemness and differentiation of BMSCs with single-cell transcriptomic analyses.

### Heterogeneous Cx43 expression associated with the stemness and differentiation of BMSCs

**Figure 5:**
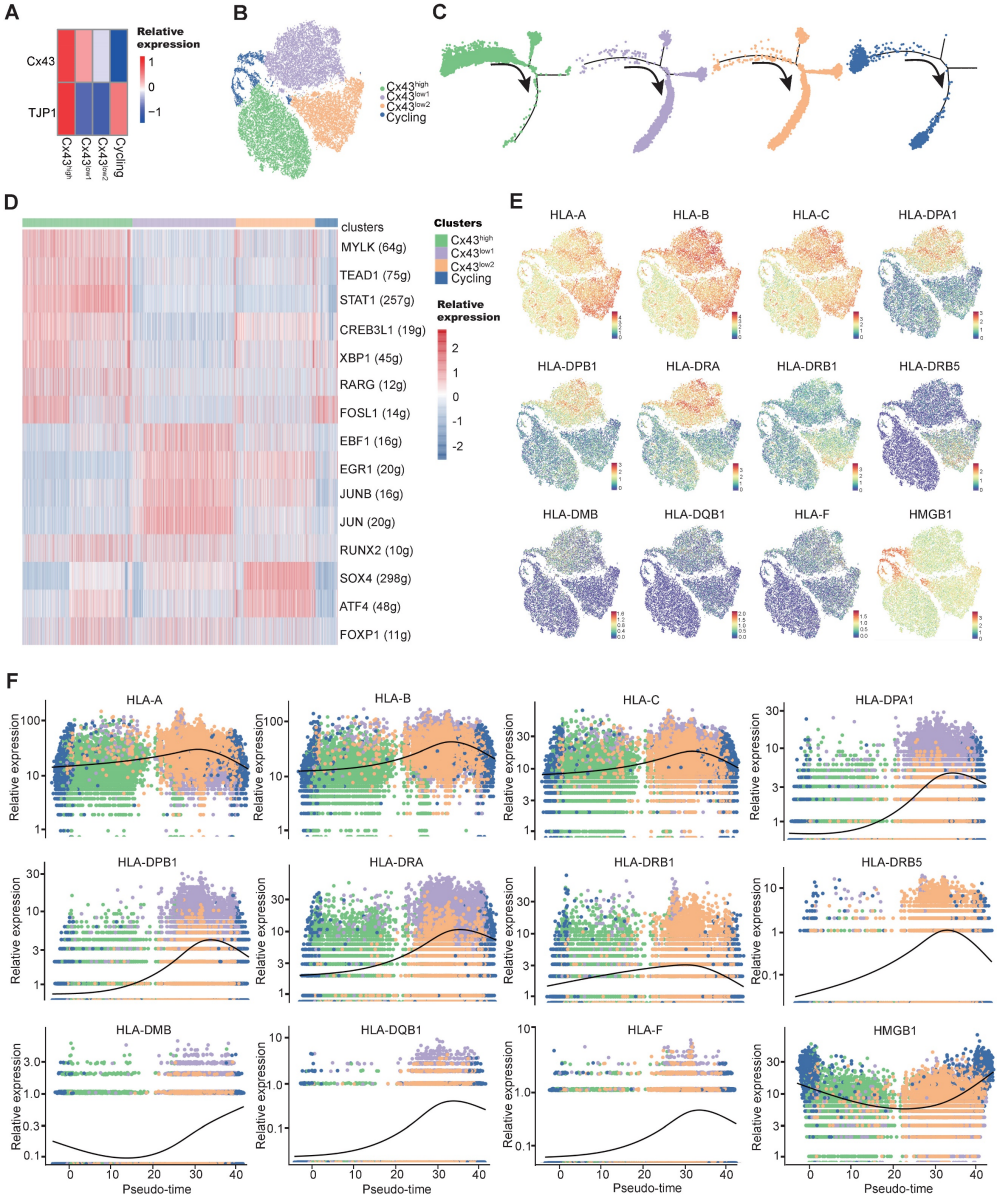
** Single-cell transcriptome exploring the connections between MSC stemness and Cx43. a** Heatmap of marker genes related to gap-junction in each BMSC subtype. **b** tSNE plot showing MSC subtypes. Each subtype was annotated according to the relative Cx43 expression, in addition to cycling BMSCs. **c** Pseudotime ordering of each MSC subtype. **d** Heatmap showing the active gene-regulatory networks across MSC subtypes predicted by the SCENIC package. **e** tSNE plot of HLA-related marker gene expression in BMSCs.** f** Dynamics of gene expression along the pseudotime. The bold line indicated mean expression across pseudotime.

To characterize the heterogeneous Cx43 expression, we have recently tracked those subclonal evolution pathways by establishing the atlas of single-MSC transcriptomes across multiple tissues and identified extracellular-matrix associated genes (ECM) associated with the potential of highly contributing to MSC heterogeneity 56. We found that ECM fibronectin can regulate Cx43 expression, and GJ Cx43 can control the production of ECM. To deepen the understanding of Cx43 functions in the heterogeneity (subclonal evolution) of BMSCs, we further depicted our recent single-MSC transcriptomes from BMSCs 56. After unsupervised clustering, the expression of subset biomarkers related to gap junctions was first identified. We found that GJ-associated proteins such as Cx43 highly expressed heterogeneously in specific subsets of BMSC subclones (Figure 5A). To describe the characterization of BMSC subclonal subsets better, non-cycling subsets were named according to their relative expression of Cx43 (Figure 5B). To further study the developmental trajectory of these BMSC subsets, pseudo time analysis was performed to order BMSC subsets along lineage-based “tree precisely.” Pseudotime inference showed that the Cx43^high^ subset was mainly located at the upstream range of the lineage tree, while the majority of two Cx43^low^ subsets were located downstream of the lineage tree, suggesting that the Cx43^high^ subset had a higher stemness capacity, compared to two Cx43^low^ subsets (Figure 5C). To infer subset-specific transcriptional regulatory networks systematically, transcriptional factors (TF) based regulons were identified using the SCENIC package, according to motif enrichment and co-expression (Figure 5D). Several subset-specific regulons were identified and potentially co-activated in either Cx43^high^ or two Cx43^low^ subsets (Figure 5D and Figure S7A, B). We observed that the regulons contributing to the maintenance of adult stem cells were active in the Cx43^high^ subset, such as TEAD1 and RARG.

In contrast, the regulons related to lineage differentiation were strongly activated in either the Cx43^ low1^ or Cx43^ low2^ subset (Figure 5D). For instance, EBF1, ATF4, and SOX4 play critical roles in promoting adipogenic, osteoblastic, and chondrogenic differentiation of MSCs, respectively. Furthermore, analysis of protein-protein interaction (PPI), subset biomarkers, secreted factors, and pathway enrichment also demonstrated that these 3 subsets were potentially functionally heterogeneous (Figure S8, Figure S9A-C, and Figure S7C). Interestingly, we observed that many human leukocyte antigens (HLA) involving in major histocompatibility complexes (MHC)-mediated antigen processing & presentation exhibited lower expression in the Cx43^high^ subset, compared to two Cx43^ low2^ subsets (Figure 5E). Besides, the expression of HLAs increased along the pseudo-time axis (Figure 5F), suggesting the low expression levels of HLAs in BMSCs were potentially linked to self-renewal. Our finding is consistent with the previous study that indicated embryonic and tissue stem cells are characteristics of HLA downregulation, and HLA-negative cancer cells possess stem-like properties 57. Interestingly, we found that its clinical relevance manifested in the levels of HLAs were significantly higher in AML patients than normal individuals in an independent sample cohort (TCGA-AML vs. GTEx) (Figure S10), suggesting their potential roles in leukemia development.

MSCs could also autonomously downregulate HLA expression through endocytosis in the feedback of the BM microenvironment 58. Our results indicated that overexpression of either miR-130a or miR-130b for targeting Cx43 could obviously increase the expression of HLAs in BMSCs (Figure S9D). Altogether, these results suggested that Cx43 potentially maintained BMSCs with self-renewal properties and reduced MSC immunogenicity. However, miR-130a/b overexpression could disturb these MSC properties. Next, we wanted to know the balanced effects of miR-130a/b on the immunosuppressive property of BMSCs.

### miR-130a/b promotes the immunosuppressive property of BMSCs

**Figure 6:**
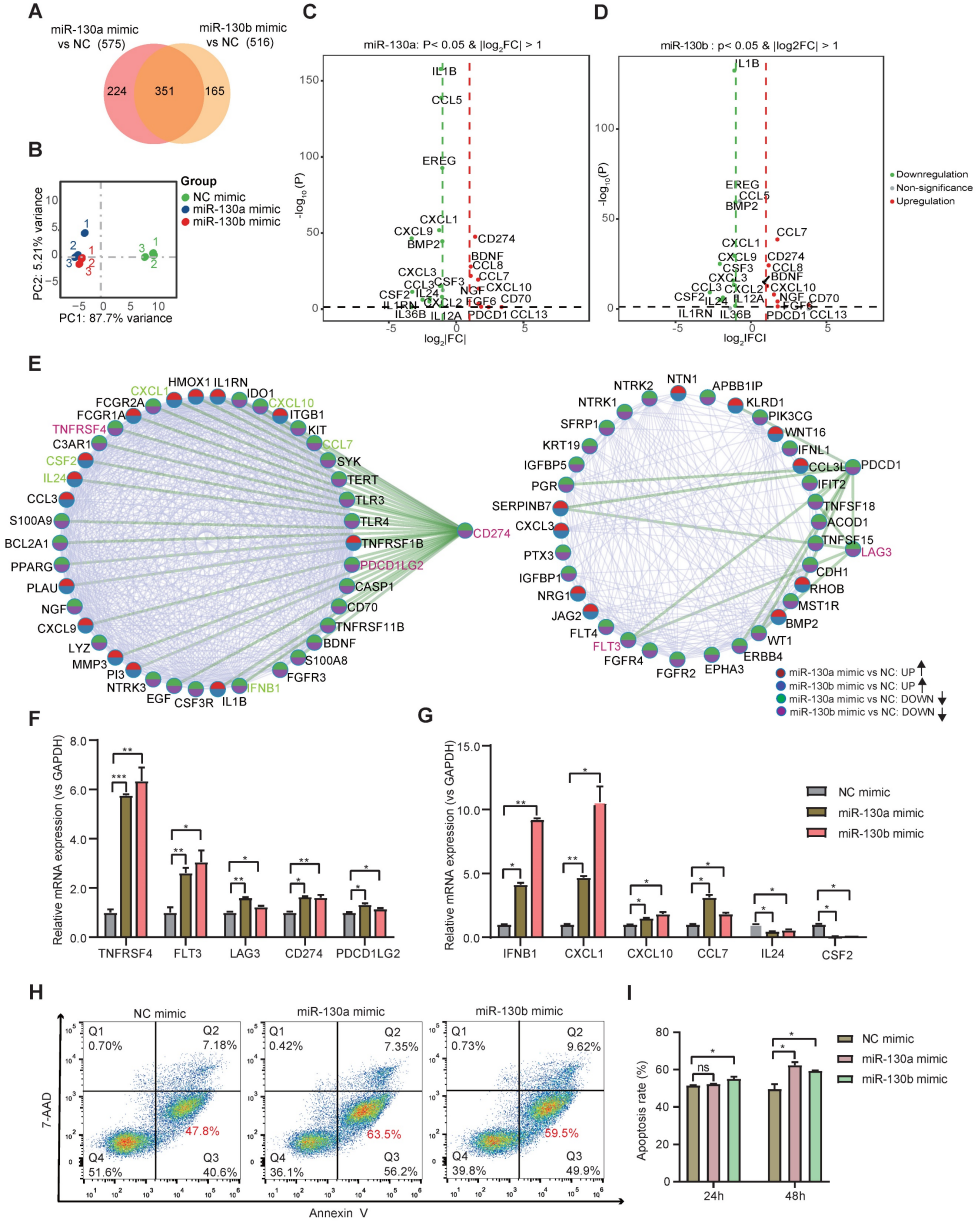
** miR-130a/b increased the immunosuppressive capacity of BMSCs. a** Venn diagram of DEGs in miR-130a and miR-130b overexpressed BMSCs. **b** Principal-component analysis (PCA) plot on the transcriptome to visualize the similarity among all the samples. **c, d** Volcano plots showing representative DEGs related to immune responses in BMSCs after miR-130a or miR-130b overexpression, respectively. **e** Protein-protein interaction network of DEGs involved in KEGG immune-related pathways. **f, g** The indicated immune checkpoint genes (**f**) and inflammatory-related genes (**g**) are assessed by Realtime PCR in BMSCs transfected with miR-130a, or miR-130b mimics at 48 hs after transfection. The relative expression levels of these genes were normalized by the NC group and GAPDH expression. Mean ± SD represents 3 independent experiments. **h, i** BMSCs were transfected with miR-130a/b mimics or NC prior to co-culture with 1x10^5^ activated CIK (CD3^+^CD56^+^) in the indicated amounts. After 24 h and 48h of co-cultures, Representative flow cytometry plots of the apoptosis marker Annexin-V and 7-AAD staining of CIK (**h**), the apoptosis percentages of CIK cells were assessed by flow cytometry(n=3) (**i**).

Bulk transcriptome profiles were applied to identify further molecular changes of BMSC's state transition by miR-130a/b overexpression. Venn diagram showed that the majority of differentially expressed genes (DEGs) overlapped between the overexpressed miR-130a and miR-130b BMSCs (Figure 6A). Principal component analysis (PCA) also indicated similar transcriptome profiles in the overexpressed miR-130a and miR-130b BMSCs (Figure 6B). Many genes associated with immune responses, such as cytokines and immunomodulation, were significantly differentially expressed after miR-130a and miR-130b overexpression in BMSCs (Figure 6C, D). For example, several immune checkpoint proteins and pathways contributing to immune escape in tumors were overexpressed after either miR-130a, or miR-130b overexpression, such as CD274, LAG3, PDCD1, and TNFRSF4 (Figure 6E). Gene ontology analysis of DEGs also showed that many pathways related to immune responses were enriched in top-20 KEGG pathways, such as cytokine-cytokine receptor interaction and PI3K-AKT pathways (Figure S11 and Figure S12A, B). Within these DEGs potentially targeted by miR-130a and miR-130b, several genes such as EGR3, TNFRSF1B, and NDRG2 also have regulatory roles in immune responses (Figure S12C). Moreover, protein-protein interaction analysis showed that these immune checkpoint proteins had a strong connection with many other DEGs (Figure 6E), suggesting that miR-130a/b systematically disturbed the immunomodulation of BMSCs. Realtime PCR further validated that the representative immune checkpoint proteins and leukemic-associated inflammatory factors, such as IFNB1, CXCL1, CXCL10, and CCL7, were significantly increased in BMSCs by either miR-130a or miR-130b overexpression, apart from IL24 and CSF2 (Figure 6F, G). To determine the contribution of miR-130a/b correlating with BMSC-induced immunosuppression, cytokine-induced killer (CIK) cells were co-cultured with BMSCs. The results suggested that the overexpression of miR-130a/b promoted the MSC-mediated immunosuppression of CIK cells (Figure 6H, I). Furthermore, inhibition of miR-130a/b could reduce the immunosuppressive ability of MSCs (Figure S13).

### BCR-ABL1 positively regulates the expression of miR-130a/b through transcription factor C/EBPβ

**Figure 7:**
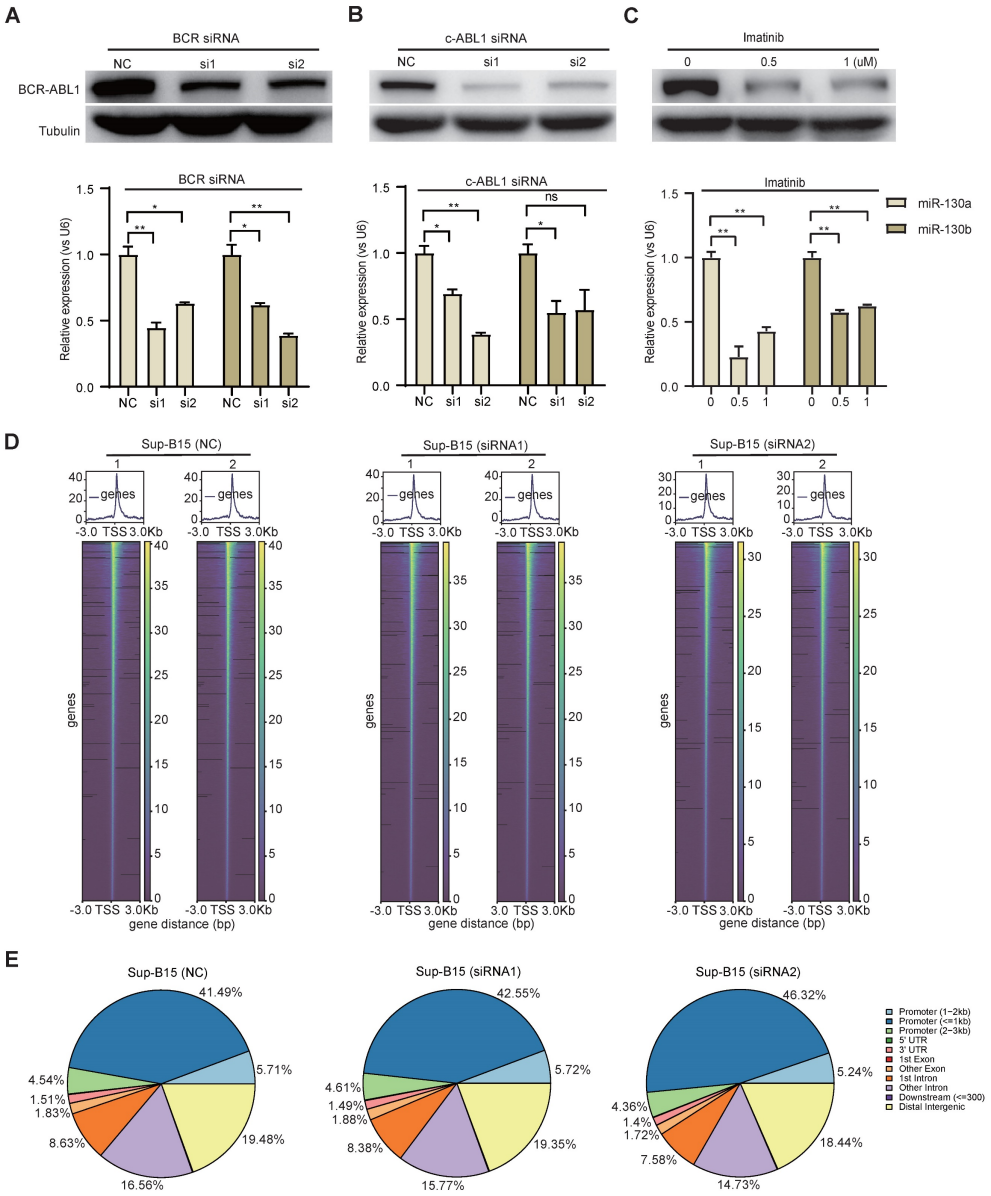
** BCR-ABL1 positively regulates the expression of miR-130a/b through transcription factor C/EBPβ. a-c** Western blot analysis of the inhibition of BCR-ABL1 protein in Sup-B15 transfected with BCR siRNA (**a**), c-ABL siRNA (**b**) and treated with imatinib (**c**) (0.5uM and 1uM) for 72h, respectively. qRT-PCR analysis of miR-130a/b in Sup-B15 treated by BCR siRNA, c-ABL siRNA, and imatinib in Sup-B15. Mean ± SD represents 3 independent replicates in all the Real-time PCR experiments. *P < 0.05, **P < 0.01, ***P < 0.001, by student's unpaired t-test. **d** Heatmap of ATAC-seq data suggesting a few differentially accessible chromatin regions between WT BMSCs and miR-130a or miR-130b overexpressing BMSCs. **e** Piechart showing the proportion of total peaks in the indicated regions.

**Figure 8:**
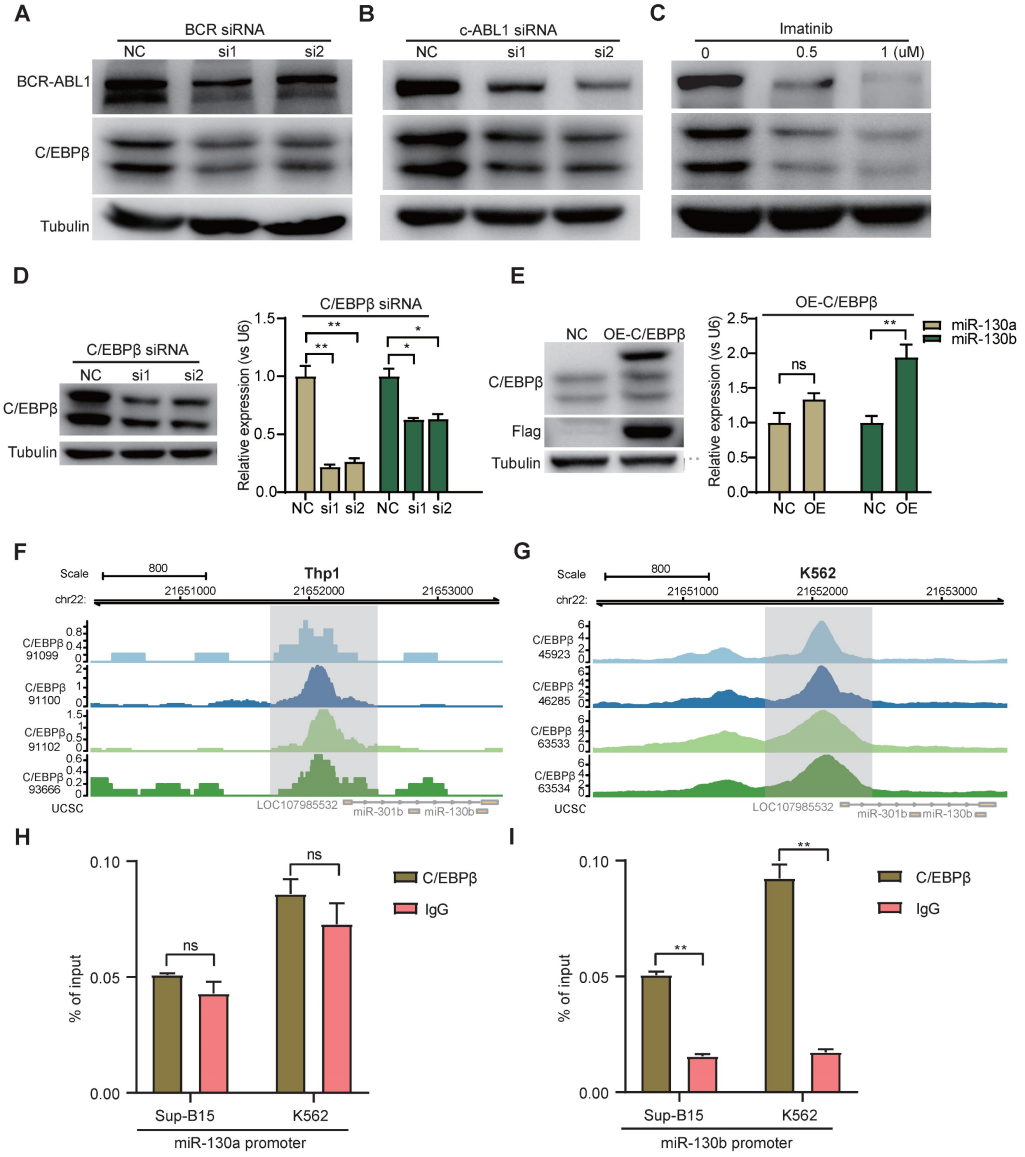
** BCR-ABL1 positively regulates the expression of miR-130a/b through transcription factor C/EBPβ. a-c** Protein levels of C/EBPβ after inhibition of BCR-ABL1 expression by BCR siRNA (**a**), c-ABL siRNA (**b**), and imatinib (**c**) in Sup-B15 for 72h, respectively. **d** Transfection of C/EBPβ siRNA knocked down C/EBPβ expression in Sup-B15. The expression of miR-130a/b was assessed in Sup-B15 transfected with C/EBPβ siRNA. **e** Sup-B15 were transduced with lentiviruses expressing C/EBPβ or control (Ctrl); Western blotting analysis identified that C/EBPβ was overexpression in Sup-B15(n=3). The expression of miR-130a/b was assessed in Sup-B15 overexpressing C/EBPβ (n=4). **f, g** Visualization of C/EBPβ ChIP-seq peaks overlapping with miR-130b promoter region in Thp1 (**f**) and K562 (**g**). **h, i** CHIP-qPCR was further validated for specific regions for both miR-130a and miR-130b promoters. Mean ± SD represents 2 independent experiments. *P < 0.05, **P < 0.01, ***P < 0.001, by student's unpaired t-test.

To study the functional consequence of the association between BCR-ABL1 and miR-130a/b, we inhibited its enzymatic activity or knockdown of BCR-ABL1 in Sup-B15 and K562 cells, respectively. The results showed that the reduction in BCR-ABL1 proteins using inhibitor *Imatinib* treatment or siRNA-mediated knockdown by BCR siRNAs or ABL1 siRNAs could significantly decrease miR-130a and miR-130b expression (Figure 7A-C and Figure S14A-C). These results suggested that the regulation of miR-130a and miR-130b expression is BCR-ABL1-dependent. To further investigate how BCR-ABL1 influenced internal signaling pathways, we employed ATAC-seq to interrogate the site-specific chromatin accessibility of BCR-ABL1 knockdown in both Sup-B15 and K562 cells, respectively. The data showed similar heatmap patterns and genomic distribution of peaks on chromatin accessibility between negative control and siRNA-mediated BCR-ABL1 knockdown in both Sup-B15 and K562 cells (Figure 7D-E and Figure S14D-E). Previous reports demonstrated that the C/EBPβ protein could be upregulated by BCR-ABL1 through STAT5 activation in CML cells, contributing to the signaling transduction of BCR-ABL1^59^. Consistently, the reduction of BCR-ABL1 mediated by knockdown using siRNAs targeting BCR and c-ABL1 or inhibitor Imatinib treatment could decrease the level of C/EBPβ protein in Sup-B15 and K562 cells (Figure 8A-C and Figure S15A-C). Furthermore, C/EBPβ knockdown also impaired the expression of miR-130a and miR-130b, while overexpression of C/EBPβ could increase miR-130a and miR-130b expression (Figure 8D-E and Figure S15D-E). These results suggested BCR-ABL1 induces miR-130a/b expression through the upregulation of C/EBPβ.

We next compiled published chromatin immunoprecipitation sequencing (ChIP-seq) data of C/EBPβ in both THP1 and K562 cells. ChIP-seq profiling showed high binding peaks in the promoter region of miR-130b and low peaks in the promoter region of miR-130a (Figure 8F-G and Figure S15F-G). Binding affinity in these two regions was further confirmed by ChIP-qPCR (Figure 8H-I). Taken together, these results established that C/EBPβ was one potential direct trans-activator of miR-130b promoter, not for miR-130a, whereas the detailed mechanisms underlying the regulation of miR-130a by C/EBPβ are still elusive.

## Discussion

In this study, we demonstrate that C/EBPβ upregulates BCR-ABL1-driven miRNA-130a/b, which could interfere with the GJIC function of BMSCs in BME by inhibiting Cx43 expression. In addition, we discover that Cx43 may maintain the self-renewal and low immunogenicity of BMSCs. This association may contribute to immunosuppressive LME. Thus, we establish the connection between exosome-regulating GJICs and LME reprogramming, indicating the functions of BCR-ABL1-driven miRNAs in the pro-leukemic training of BMSCs. We realize we did not have sufficient clinical verification to translate the knowledge to manage AML and ALL cancer resistance via regulating BCR-ABL1-driven miRNAs.

Nonetheless, our principal results are supported by increasing evidence showing that BMSCs can enhance drug resistance, proliferation, and quiescence of leukemia cells via exosomes and direct intercellular interaction 15. New therapeutics can be developed by identifying signaling pathways within LME's intercellular communication networks. In clinical trials, blocking immune checkpoint regulatory proteins or preventing the communication of tumor cells to their specific niches has shown promising results 60.

The study of BMSCs from patients with blood cancer and healthy individuals reveals differences in morphology, differentiation capacity, and growth rate of BMSCs, in addition to that, we 25 and others 61 show the altered GJIC functions in BMSCs generated from AML patients, including the decrease in GJIC-Cx43 protein. Cx43, as an integral membrane protein, generates gap junction channels, which mediate intracellular signaling and intercellular transport in the microenvironment. When Cx43 is overexpressed in BMSCs, it reduces leukemia cells' anti-drug resistance, enhancing AML apoptosis 27. The peripheral blood blast rate in leukemia cells negatively correlates with Cx43 expression 62. Furthermore, increased expression of Cx43 triggers mature differentiation of AML cells, which promotes leukemia remission 63. All the studies suggested that Cx43 has suppressive roles in leukemogenesis in LME. However, in many types of solid tumors, Cx43 could act as a tumor suppressor to inhibit carcinogenesis or as an oncogene to induce cancer metastasis. Connexins such as Cx43 generally facilitate rather than block cancer metastasis 64. However, whether Cx43 involves BMSCs in leukemic infiltration and transformation is still unknown.

We fill the gap between leukemic infiltration and transformation by linking Cx43-involved BMSCs dots to miR-130a/b overexpression. MiRNAs in exosomes generated from leukemic cells govern leukemogenesis and relapse, and miR-130a/b overexpression has been observed to increase different types of leukemogenesis and therapy resistance. For instance, miR-130a overexpression in AML is associated with greater etoposide resistance 42. In children, acute promyelocytic leukemia (APL) is accelerated by miR-130b overexpression targeting PTEN 46.

As a constitutive kinase that also possesses tyrosine kinase activity, BCR-ABL1 is responsible for regulating many miRNAs that play a role in the development of leukemia. In cases of chronic myeloid leukemia (CML), BCR-ABL1 inhibited the expression of miR-150, which contributed to a halt in myeloid differentiation and treatment resistance 65. BCR-ABL1 upregulates the expression of the miR-17-92 cluster, which facilitates the leukemogenesis of CML 66. We initially demonstrated that the oncogene BCR-ABL1 increases miR-130a/b expression via C/EBP in both B-ALL and CML cells. In addition, leukemia cells secrete exosomes associated with their educated stromal cells, revealing the molecular identities and characteristics of LME. Our data suggest that BCR-ABL1-driven miRNA-130a/b might be transferred into adjacent BMSCs by exosomes, hence enhancing their immunosuppressive properties and adipogenic potential.

Leukemic treatment resistance may come from senescence triggered by adhesion molecules and related mechanical pressure. The Cx43-ablated cells exhibit more senescent phenotypes, including the failure of proper differentiation *in vitro,* and Cx43 in HSCs acts as a defense mechanism against senescence by releasing reactive oxygen species into the microenvironment 67. Furthermore, Cx43 can delay senescence and maintain stemness properties of induced MSCs derived from human induced pluripotent stem cells (iPSC) 68. Our results suggested that Cx43^high^ BMSCs exhibited a higher stemness capacity than Cx43^low^ BMSCs (Figure 5C). This is consistent with a previous report 69, as well as that the stemness marker genes (OCT4, SOX2, and NANOG) were significantly decreased after siRNA targeting Cx43^70^. We also observed that a deficiency of Cx43 could induce adipogenesis and delay osteogenic regeneration (Figure 4E, F). In LME, many studies suggested that increased adipocytes derived from MSC adipogenesis could preserve the survival of leukemia cells by altering energy metabolism and fatty acid oxidation 71. Furthermore, adipocytes contribute to the pro-inflammatory niches for leukemia cells 55, which cause DNA damage and lead to carcinogenesis 72. Synthetic cell adhesion molecules' modularity illuminates how differentcell-cell interface classes may have originated, inferring that it is possible to generate a wide variety of synthetic cell adhesion molecules by combining orthogonal extracellular interactions with intracellular domains derived from native adhesion molecules for heterotypic gap junctions on tumor cells and immune cells 73. The therapeutic potential of modified adhesion molecules includes the precise guidance of tissue repair and regeneration and regulating the contacts and trafficking of immune and cancer cells.

Resistance to leukemic therapy also manifests itself in transcriptional regulation, as indicated in our data and supported by the literature. Transcription factor C/EBPβ, an essential regulator of the CAAT/enhancer-binding protein family, regulates the proliferation and differentiation of myeloid progenitors 74. The upregulation of C/EBPβ induced by BCR-ABL1 was found in EML cells (a mouse HSC line) 75. Furthermore, the existence of their enhancers collaborates with upregulated C/EBPβ to induce myeloid leukemia 59. C/EBPβ transcriptional network contributed to B-ALL leukemogenesis 76. However, C/EBPβ was also reported to exert opposite effects and was negatively regulated by BCR-ABL1 77. The induction of C/EBPβ activity could trigger ATRA-induced maturation of acute promyelocytic leukemia, contributing to the exhaustion of leukemia cells 78. Here, we found that BCR-ABL1 promotes the expression of C/EBPβ (Figure 8A-C and Figure S16A-C), which increased the expression of miRNA-130a/b. There were binding regions for C/EBPβ on the miR-130b promoter but not on the miR-130a promoter (Figure 8F-G and Figure S16F-G), suggesting that C/EBPβ may indirectly induce the expression of miRNA-130a.

In conclusion, our research help to clarify how leukemogenesis is facilitated by BCR-ABL1-mediated Cx43 signaling transduction within BMME. To stop the modification of the BMME caused by BCR-ABL1-driven exosomes, new treatment modalities may be considered for heterotypic gap junctions on tumor cells and immune cells. This action potentially leads to subclonal evolution of therapeutic resistance, a mechanism by which cancer progression and posttreatment recurrence by subclonal switchboard signaling shift the dormant subclones to dominating subclones, as previously described 3. Overall, these findings could have implications for understanding the development and progression of leukemia with the BCR-ABL1 fusion gene, as well as potential targets for therapeutic interventions.

## Methods

### Clinical patient samples

The bone marrow aspirates and peripheral blood specimens were obtained from newly-diagnosed CML and B-ALL patients at the Second Affiliated Hospital, Army Medical University, between 2017 and 2020. The detection of BCR-ABL1 mRNA expression was assessed by Real-time PCR assays using BCR-ABL1 P210 or P190 One-Step Detection Kit (Yuanqi Bio, China; CA10007), following the manufacturer's manual. Normal bone marrow specimens were collected from healthy donors for hematopoietic stem cell transplantation (HSCT). Following the previous report, each sample's plasmas and peripheral blood mononuclear cells were isolated 79. All the related procedures were approved by the internal review and ethics boards from our hospitals. Relative informed consent was obtained from all the patients or healthy donors, which complied with all applicable ethical regulations.

### Cell culture

All leukemic and HEK293T cell lines were purchased from the Type Culture Collection of the Chinese Academy of Sciences (Shanghai, China). Leukemic HL60, K562, BALL-1, and Jurkat cells (ATCC) were expanded in RPMI-1640 culture medium (Gibco, 8122022), supplemented with 10% fetal bovine serum (Hyclone, S711-001S) and suitable antibiotics (penicillin and streptomycin) (1:100, Hyclone). Sup-B15 cells were cultured in Iscove's modified Dulbecco's medium (Gibco, 8121066), supplemented with 20% FBS and antibiotics. HEK293T cells were cultured in Dulbecco's Modified Eagle Medium medium (Gibco, 8122154), supplemented with 10% FBS and antibiotics.

BMSCs were isolated from bone marrow aspirations of healthy donors, following our previous report 26. The BMSC medium was replaced each 2-3 days until approximately 90% confluent. The quality of BMSCs was also determined by cellular surface markers (positive for CD90, CD73, and CD105, and negative for CD11b, CD19, CD34, CD45, and HLA‐DR) using Human MSC Analysis Kit (BD,562245) via flow cytometry (Beckman) and tri-lineage *in vitro* differentiation assays, according to our previous report 56. All cells were cultured in an incubator at 37°C with 5% CO_2_. The cells were passaged every 2 or 3 days.

### Exosomes isolation and application

The culture supernatants were collected 72 hs (h) after cells were cultured in a fresh FBS medium. The media was collected and subjected to a low-level spin at 500 × g for 5 mins to remove cells, followed by sequential centrifugation of 2000xg for 10 mins to remove dead cells, then the supernatant was collected and spun in the preparative ultracentrifuge at 10000 g for 30 min to remove cell debris. The supernatant was transferred to new ultracentrifuge bottles and ultracentrifuge for 2 h at 100,000 x g, 4°C. To wash the EV pellet, the pellet was resuspended with PBS and centrifuged for 2 h at 100,000 x g, 4°C. Finally, the supernatant was discarded and resuspended using cold 1x PBS to obtain the final EV pellet. Exosomes were also efficiently isolated from serum, plasma, and cell culture supernatant using a membrane affinity spin column, according to the manufacturer's exoEasy Maxi Kit (QIAGEN, 76064) procedure. The protein content of exosomes was quantified using BCA Protein Assay (Beyotime, P0010S). 2 × 10^5^ BMSCs were incubated with 50 μg exosomes for 48 h. Then cells were utilized for the following experiments.

### Cell transfection & Lentivirus infection

When the confluence was near 50-70%, BMSCs were transfected siRNAs (100 nM) (TSINGKE Company), miRNA mimics (100 nM), or miRNA inhibitors (150 nM) (RiboBio Company, C10712-1) with Lipofectamine RNAiMAX (Invitrogen, 2270667, USA), respectively. The detailed protocols for siRNA or miRNA transfection were performed according to the manufacturer's instructions. The detailed sequences of all the siRNAs were attached in Table S1 and Table S2. About 48 or 72 h after transfection, cells were harvested for Realtime PCR and western blotting detection. Lentiviruses for the overexpression of miR-130a or miR-130b were designed and produced by TSINGKE (Shanghai, China). The infected BMSCs were passaged every three days at a ratio of 1:3. The full-length human sequence of Cx43 was cloned into the pRuf-IRES-RFP vector. BMSCs with stable overexpression of Cx43 were enriched by fluorescence-activated cell sorting.

### Fluorescence recovery after photobleaching (FRAP) assay

BMSCs were plated on a 20-mm glass-bottomed dish (NEST, 801001) and grown to near 50% confluence. FRAP assay was performed on BMSCs stained with 10 μg/ml calcein-AM (Invitrogen, C34852) using the laser confocal microscope SP5 (Leica). Each cell was photobleached to such an extent that the ROI fluorescence was reduced by at least 20% of their initial fluorescence. Their neighbor cells were selected as relative visual control. The fluorescence recovery was monitored using images taken after photobleaching for 152 seconds at an interval of 48 seconds (s). The speed of fluorescence recovery was calculated according to previous reports 80.

### Western blotting

Cells or exosomes were lysed with a lysis buffer containing a complete protease inhibitor (Beyotime, China). The western blotting protocol was detailed in our prior reports 56. Antibodies dilutions used were rabbit anti-human GJA1 (1:4000, Abcam, ab11370, USA), C/EBPβ (1:500, Santa Cruz, sc-7962, USA), c-Abl (1:1000, CST, 2862T, USA), TSG101 (1:8000, Proteintech, 28283-1-AP, China), Calnexin (1:20000, Proteintech, 10427-2-AP, China), CD9 (1:3000, Proteintech, 20597-1-AP, China), and α-TUBULIN (1:10000, Proteintech, 11224-1-AP, China). The HRP-conjugated secondary antibody (Proteintech, Wuhan, China) was diluted into 1:10000. Western blots were quantified by densitometry using the ImageJ software. All experiments were normalized by α-TUBULIN expression.

### RNA extraction and quantitative real-time PCR

Cellular or exosomal RNA was isolated using TRIzol reagent (Invitrogen) or miRCURRY LAN miRNA PCR Starter Kit (QIAGEN, 339320). Total RNA was reverse transcribed into cDNA using a cDNA Reverse Transcription Kit (TaKaRa, RR047A), according to the manufacturer's procedure. Real-time PCR was performed using Quantitative TB green (TaKaRa, RR820A) in triplicate with ABI 7500 fast real-time PCR System (Applied Biosystems, USA). miRNA expression assays were generated using miRNA-specific primers mixes (TSINGKE, China) and miRNA first stranded synthesis Kit (Sangon Biotech, B532451). The expression of miRNAs and coding genes was calculated through normalization to U6 or Actin as internal controls, respectively. The detailed sequences of all the primers are attached in Supplementary Table 1.

### Luciferase reporter assay

293T cells were co-transfected with psiCHECK2 luciferase plasmids containing wild-type or mutant 3'-UTR of GJA1(1ug, TSINGKE Company) with miR-130a-3p mimics, miR-130b-3p mimics or mimic-NC (150nM, RiboBio Company) using Lipofectamine 3000 (Invitrogen), respectively. Luciferase activity was measured 48h after transfection using the Dual-Glo® Luciferase Assay System (Promega, E2940). All the experiments were repeated three times, in triplicate each time.

### Chromatin immunoprecipitation (ChIP) assay and ChIP-qPCR

ChIP assay was performed using the Simple ChIP Enzymatic Chromatin IP Kit (Cell Signaling Technology, USA), according to the manufacturer's instructions. Briefly, 1×10^7^ Sup-B15 or K562 cells were fixed with 1% formaldehyde for 10 min (minutes) at room temperature. Fixed cells were lysed and sonicated using a sonicator (Shangchao, FS-250N, China) and pulsed for 5 cycles of 20 s on with 30 s off. 20ul Anti- C/EBPβ (200 ng/ul, Santa Cruz, sc-7962, USA) and 10 ul anti-mouse IgG (400 ng/ul, Santa Cruz, sc-2025) were used for immunoprecipitation. The immuno-precipitated DNA was subjected to Realtime PCR using specific primer pairs to detect the indicated regions. The primer sequences used in Realtime PCR after ChIP assays are listed in Table S**1**. Experiments were independently repeated more than three times.

### Lineage differentiation of BMSCs *in vitro*

Osteogenic and adipogenic differentiation assays were performed using an osteogenic induction medium (Cyagen, HUXMA-90021) and an adipogenic induction medium (Cyagen, HUXMA-90031). The detailed protocols were performed according to our previous reports 56.

### Co-Culture experiments

Six-well plates were filled with transwell chambers (0.4 um pore size, Corning Incorporated, NY, USA). were seeded. When BMSCs grew to 90% of confluence in the lower chamber, leukemia cells (2x10^6^) were placed in the upper chamber. BMSCs were harvested after 48 hs of co-culture with leukemia cells. For CIK co-culture experiments, CIK cells were first activated with Recombinant Human IL2 (200U/mL, Abcam). 5x10^5^ activated CIK cells were loaded into the upper chamber, and BMSCs (5x10^5^) were seeded in the lower chamber. After 24 or 48 hs of co-culture, CIK cells were harvested for apoptosis detection by flow Cytometry.

### Flow cytometry

BMSCs were resuspended in PBS containing 1% FBS and stained with a fluorescent-conjugated positive cocktail (CD73, CD90, CD105) and negative cocktail (CD11b, CD19, CD34, CD45, HLA-DR) using Human MSC Analysis Kit (BD,562245). The appropriate isotype control was set as a negative control, according to the manufacturer's instructions. The vitality of CIK cells was analyzed using an Annexin V-APC Apoptosis Detection Kit (BioLegend, 640930), according to the manufacturer's instructions.

### RNA isolation and library preparation for bulk transcriptome profiling

Total RNA from BMSCs was extracted using the TRIzol reagent (Thermo Fisher, USA), according to the manufacturer's protocol. The NanoDrop 2000 spectrophotometer was further used to evaluate RNA purity and quantification (Thermo Scientific, USA). At the same time, the Agilent 2100 Bioanalyzer was used to assess RNA integrity (Agilent Technologies, Santa Clara, CA, USA). The high quality and integrity of total RNAs were performed to prepare transcriptome libraries. The sequencing libraries were constructed using TruSeq Stranded mRNA LT Sample Prep Kit (Illumina, San Diego, CA, USA) according to the manufacturer's instructions. After that, these libraries were sequenced on an Illumina HiSeq x Ten platform using 150-bp paired-end sequencing.

### Statistical information

All the images were digitally processed and labeled using Image-Pro Plus and Illustrator CC 2019. Statistical analyses were performed using GraphPad Prism 8 software. Two-tailed paired or unpaired student's t-tests were used to determine statistical significance when comparing two groups. The value of p < 0.05, 0.01, and 0.001 was set for the thresholds of statistical significance. Data are shown as the mean ± SEM. For bioinformatics analysis on next-generation sequencing data, detailed information was seen in Supplementary Methods.

### NGS bioinformatics analysis

All the high-quality sequencing data were analyzed following the standard or customized pipelines. The detailed information was described in Supplementary information.

## Supplementary Material

Supplementary methods and figures.

## Abbreviations

ALL: acute lymphoblastic leukemia
APL: acute promyelocytic leukemia
B-ALLs: B progenitor acute lymphoblastic leukemia
BMME: bone marrow microenvironment
CML: chronic myeloid leukemia
GJICs: gap-junction intercellular communications
HLA: human leukocyte antigens
HSPCs: hematopoietic stem/progenitor cells
LME: leukemic microenvironment
MSCs: Mesenchymal stem/stromal cells
TKI: tyrosine kinase inhibitors

## References

[1] von Bubnoff N, Schneller F, Peschel C, Duyster J (2002). BCR-ABL gene mutations in relation to clinical resistance of Philadelphia-chromosome-positive leukaemia to STI571: a prospective study. Lancet.

[2] Cheson BD (2012). Clinical advances in hematology & oncology. Clin Adv Hematol Oncol.

[3] Li SC, Lee KL, Luo J (2012). Control dominating subclones for managing cancer progression and posttreatment recurrence by subclonal switchboard signal: implication for new therapies. Stem Cells Dev.

[4] Podvin B, Guermouche H, Roynard P, Goursaud L, Berthon C, Ouafi M (2022). Subclonal acquisition of a BCR::ABL1 fusion in a chronic myelomonocytic leukemia. Ann Hematol.

[5] Ikeda D, Chi S, Uchiyama S, Nakamura H, Guo YM, Yamauchi N (2022). Molecular Classification and Overcoming Therapy Resistance for Acute Myeloid Leukemia with Adverse Genetic Factors. Int J Mol Sci.

[6] Tachibana T, Kondo T, Uchida N, Doki N, Takada S, Takahashi S (2022). The Clinical Significance of BCR-ABL1 Mutations in Patients With Philadelphia Chromosome-Positive Chronic Myeloid Leukemia Who Underwent Allogeneic Hematopoietic Cell Transplantation. Transplant Cell Ther.

[7] Gorre ME, Mohammed M, Ellwood K, Hsu N, Paquette R, Rao PN (2001). Clinical resistance to STI-571 cancer therapy caused by BCR-ABL gene mutation or amplification. Science (New York, NY.

[8] Zhao H, Deininger MW (2020). Declaration of Bcr-Abl1 independence. Leukemia.

[9] Balabanov S, Braig M, Brummendorf TH (2014). Current aspects in resistance against tyrosine kinase inhibitors in chronic myelogenous leukemia. Drug Discov Today Technol.

[10] Ma D, Liu P, Wang P, Zhou Z, Fang Q, Wang J (2021). PKC-beta/Alox5 axis activation promotes Bcr-Abl-independent TKI-resistance in chronic myeloid leukemia. J Cell Physiol.

[11] Tabe Y, Jin L, Iwabuchi K, Wang RY, Ichikawa N, Miida T (2012). Role of stromal microenvironment in nonpharmacological resistance of CML to imatinib through Lyn/CXCR4 interactions in lipid rafts. Leukemia.

[12] Warsch W, Walz C, Sexl V (2013). JAK of all trades: JAK2-STAT5 as novel therapeutic targets in BCR-ABL1+ chronic myeloid leukemia. Blood.

[13] Chu S, Li L, Singh H, Bhatia R (2007). BCR-tyrosine 177 plays an essential role in Ras and Akt activation and in human hematopoietic progenitor transformation in chronic myelogenous leukemia. Cancer Res.

[14] Zhang X, Hou L, Li F, Zhang W, Wu C, Xiang L (2022). Piezo1-mediated mechanosensation in bone marrow macrophages promotes vascular niche regeneration after irradiation injury. Theranostics.

[15] Shah M, Bhatia R (2018). Preservation of Quiescent Chronic Myelogenous Leukemia Stem Cells by the Bone Marrow Microenvironment. Advances in experimental medicine and biology.

[16] Mudry RE, Fortney JE, York T, Hall BM, Gibson LF (2000). Stromal cells regulate survival of B-lineage leukemic cells during chemotherapy. Blood.

[17] Borriello L, Nakata R, Sheard MA, Fernandez GE, Sposto R, Malvar J (2017). Cancer-Associated Fibroblasts Share Characteristics and Protumorigenic Activity with Mesenchymal Stromal Cells. Cancer Res.

[18] Gomzikova MO, Zhuravleva MN, Vorobev VV, Salafutdinov, II, Laikov AV, Kletukhina SK (2019). Angiogenic Activity of Cytochalasin B-Induced Membrane Vesicles of Human Mesenchymal Stem Cells. Cells.

[19] Buggins AG, Milojkovic D, Arno MJ, Lea NC, Mufti GJ, Thomas NS (2001). Microenvironment produced by acute myeloid leukemia cells prevents T cell activation and proliferation by inhibition of NF-kappaB, c-Myc, and pRb pathways. J Immunol (Baltimore, Md: 1950).

[20] Ciciarello M, Corradi G, Forte D, Cavo M, Curti A (2021). Emerging Bone Marrow Microenvironment-Driven Mechanisms of Drug Resistance in Acute Myeloid Leukemia: Tangle or Chance?. Cancers.

[21] Cancelas JA, Koevoet WL, de Koning AE, Mayen AE, Rombouts EJ, Ploemacher RE (2000). Connexin-43 gap junctions are involved in multiconnexin-expressing stromal support of hemopoietic progenitors and stem cells. Blood.

[22] Montecino-Rodriguez E, Leathers H, Dorshkind K (2000). Expression of connexin 43 (Cx43) is critical for normal hematopoiesis. Blood.

[23] Nguyen TD, Taffet SM (2009). A model system to study Connexin 43 in the immune system. Mol Immunol.

[24] Leithe E, Sirnes S, Omori Y, Rivedal E (2006). Downregulation of gap junctions in cancer cells. Crit Rev Oncog.

[25] Liu Y, Zhang X, Li ZJ, Chen XH (2010). Up-regulation of Cx43 expression and GJIC function in acute leukemia bone marrow stromal cells post-chemotherapy. Leukemia Res.

[26] Zhang X, Liu Y, Si YJ, Chen XH, Li ZJ, Gao L (2012). Effect of Cx43 gene-modified leukemic bone marrow stromal cells on the regulation of Jurkat cell line *in vitro*. Leukemia Res.

[27] Yang S, Wen Q, Liu Y, Zhang C, Wang M, Chen G (2015). Increased expression of CX43 on stromal cells promotes leukemia apoptosis. Oncotarget.

[28] McLachlan E, Shao Q, Wang HL, Langlois S, Laird DW (2006). Connexins act as tumor suppressors in three-dimensional mammary cell organoids by regulating differentiation and angiogenesis. Cancer Res.

[29] Dominiak A, Chełstowska B, Olejarz W, Nowicka G (2020). Communication in the Cancer Microenvironment as a Target for Therapeutic Interventions. Cancers.

[30] Chen Q, Boire A, Jin X, Valiente M, Er EE, Lopez-Soto A (2016). Carcinoma-astrocyte gap junctions promote brain metastasis by cGAMP transfer. Nature.

[31] Zhang HC, Zhang ZS, Zhang L, Wang A, Zhu H, Li L (2018). Connexin 43 in splenic lymphocytes is involved in the regulation of CD4+CD25+ T lymphocyte proliferation and cytokine production in hypertensive inflammation. Int J Mol Med.

[32] Hofmann F, Navarrete M, Álvarez J, Guerrero I, Gleisner MA, Tittarelli A (2019). Cx43-Gap Junctions Accumulate at the Cytotoxic Immunological Synapse Enabling Cytotoxic T Lymphocyte Melanoma Cell Killing. Int J Mol Med.

[33] Neijssen J, Pang B, Neefjes J (2007). Gap junction-mediated intercellular communication in the immune system. Prog Biophys Mol Biol.

[34] Li SC, Kabeer MH (2022). Caveolae-Mediated Extracellular Vesicle (CMEV) Signaling of Polyvalent Polysaccharide Vaccination: A Host-Pathogen Interface Hypothesis. Pharmaceutics.

[35] Sun Y, Revach OY, Anderson S, Kessler EA, Wolfe CH, Jenney A (2023). Targeting TBK1 to overcome resistance to cancer immunotherapy. Nature.

[36] Corrado C, Raimondo S, Saieva L, Flugy AM, De Leo G, Alessandro R (2014). Exosome-mediated crosstalk between chronic myelogenous leukemia cells and human bone marrow stromal cells triggers an interleukin 8-dependent survival of leukemia cells. Cancer Lett.

[37] Kumar B, Garcia M, Weng L, Jung X, Murakami JL, Hu X (2018). Acute myeloid leukemia transforms the bone marrow niche into a leukemia-permissive microenvironment through exosome secretion. Leukemia.

[38] Jafarzadeh N, Safari Z, Pornour M, Amirizadeh N, Forouzandeh Moghadam M, Sadeghizadeh M (2019). Alteration of cellular and immune-related properties of bone marrow mesenchymal stem cells and macrophages by K562 chronic myeloid leukemia cell derived exosomes. J Cell Physiol.

[39] Taverna S, Amodeo V, Saieva L, Russo A, Giallombardo M, De Leo G (2014). Exosomal shuttling of miR-126 in endothelial cells modulates adhesive and migratory abilities of chronic myelogenous leukemia cells. Mol Cancer.

[40] Alemdehy MF, de Looper HW, Kavelaars FG, Sanders MA, Hoogenboezem R, Löwenberg B (2016). MicroRNA-155 induces AML in combination with the loss of C/EBPA in mice. Leukemia.

[41] Viola S, Traer E, Huan J, Hornick NI, Tyner JW, Agarwal A (2016). Alterations in acute myeloid leukaemia bone marrow stromal cell exosome content coincide with gains in tyrosine kinase inhibitor resistance. British J Haematol.

[42] Ding C, Chen SN, Macleod RAF, Drexler HG, Nagel S, Wu DP (2018). MiR-130a is aberrantly overexpressed in adult acute myeloid leukemia with t(8;21) and its suppression induces AML cell death. Ups J Med Sci.

[43] Malouf C, Antunes ETB, O'Dwyer M, Jakobczyk H, Sahm F, Landua SL (2021). miR-130b and miR-128a are essential lineage-specific codrivers of t(4;11) MLL-AF4 acute leukemia. Blood.

[44] Li X, Xiao Y, Wang X, Huang R, Wang R, Deng Y (2023). Connexin 43-modified bone marrow stromal cells reverse the imatinib resistance of K562 cells via Ca 2+ -dependent gap junction intercellular communication. Chin Med J (Engl).

[45] Peng Z, Duan F, Yin J, Feng Y, Yang Z, Shang J (2019). Prognostic values of microRNA-130 family expression in patients with cancer: a meta-analysis and database test. J Transl Med.

[46] Gong XC, Xu YQ, Jiang Y, Guan H, Liu HL (2016). Onco-microRNA miR-130b promoting cell growth in children APL by targeting PTEN. Asian Pac J Trop Med.

[47] Gu S, Jin L, Zhang Y, Huang Y, Zhang F, Valdmanis PN (2012). The loop position of shRNAs and pre-miRNAs is critical for the accuracy of dicer processing *in vivo*. Cell.

[48] Zietzer A, Werner N, Jansen F (2018). Regulatory mechanisms of microRNA sorting into extracellular vesicles. Acta physiologica (Oxford, England). Acta Physiol (Oxf).

[49] Carter N, Mathiesen AH, Miller N, Brown M, Colunga Biancatelli RML, Catravas JD (2022). Endothelial cell-derived extracellular vesicles impair the angiogenic response of coronary artery endothelial cells. Front Cardiovasc Med.

[50] Fevrier B, Raposo G (2004). Exosomes: endosomal-derived vesicles shipping extracellular messages. Curr Opin Cell Biol.

[51] Forte D, García-Fernández M, Sánchez-Aguilera A, Stavropoulou V, Fielding C, Martín-Pérez D (2020). Bone Marrow Mesenchymal Stem Cells Support Acute Myeloid Leukemia Bioenergetics and Enhance Antioxidant Defense and Escape from Chemotherapy. Cell metabolism.

[52] Blau O, Hofmann WK, Baldus CD, Thiel G, Serbent V, Schümann E (2007). Chromosomal aberrations in bone marrow mesenchymal stroma cells from patients with myelodysplastic syndrome and acute myeloblastic leukemia. Exp Hematol.

[53] Pittenger MF, Mackay AM, Beck SC, Jaiswal RK, Douglas R, Mosca JD (1999). Multilineage potential of adult human mesenchymal stem cells. Science (New York, NY).

[54] Frisch BJ, Ashton JM, Xing L, Becker MW, Jordan CT, Calvi LM (2012). Functional inhibition of osteoblastic cells in an *in vivo* mouse model of myeloid leukemia. Blood.

[55] Ye H, Adane B, Khan N, Sullivan T, Minhajuddin M, Gasparetto M (2016). Leukemic Stem Cells Evade Chemotherapy by Metabolic Adaptation to an Adipose Tissue Niche. Cell Stem Cell.

[56] Wang Z, Chai C, Wang R, Feng Y, Huang L, Zhang Y (2021). Single-cell transcriptome atlas of human mesenchymal stem cells exploring cellular heterogeneity. Clin Transl Med.

[57] Burr ML, Sparbier CE, Chan KL, Chan YC, Kersbergen A, Lam EYN (2019). An Evolutionarily Conserved Function of Polycomb Silences the MHC Class I Antigen Presentation Pathway and Enables Immune Evasion in Cancer. Cancer Cell.

[58] Wang Y, Huang J, Gong L, Yu D, An C, Bunpetch V (2019). The Plasticity of Mesenchymal Stem Cells in Regulating Surface HLA-I. iScience.

[59] Yokota A, Hirai H, Sato R, Adachi H, Sato F, Hayashi Y (2019). C/EBPβ is a critical mediator of IFN-α-induced exhaustion of chronic myeloid leukemia stem cells. Blood Adv.

[60] Pardoll DM (2012). The blockade of immune checkpoints in cancer immunotherapy. Nature Rev Cancer.

[61] Kouzi F, Zibara K, Bourgeais J, Picou F, Gallay N, Brossaud J (2020). Disruption of gap junctions attenuates acute myeloid leukemia chemoresistance induced by bone marrow mesenchymal stromal cells. Oncogene.

[62] Fateen M, Seif A, Salama R, Shams A, Amin D (2021). The Relationship between the Connexin 32 and Connexin 43 Genes and the Pretreatment Stage and Short-term Follow-up of Patients with Acute Myeloid Leukemia. Iranian J Med Sci.

[63] Reikvam H, Ryningen A, Sæterdal LR, Nepstad I, Foss B, Bruserud Ø (2015). Connexin expression in human acute myeloid leukemia cells: identification of patient subsets based on protein and global gene expression profiles. Int J Mol Med.

[64] Aasen T, Mesnil M, Naus CC, Lampe PD, Laird DW (2016). Gap junctions and cancer: communicating for 50 years. Nature Rev Cancer.

[65] Srutova K, Curik N, Burda P, Savvulidi F, Silvestri G, Trotta R (2018). BCR-ABL1 mediated miR-150 downregulation through MYC contributed to myeloid differentiation block and drug resistance in chronic myeloid leukemia. Haematologica.

[66] Venturini L, Battmer K, Castoldi M, Schultheis B, Hochhaus A, Muckenthaler MU (2007). Expression of the miR-17-92 polycistron in chronic myeloid leukemia (CML) CD34+ cells. Blood.

[67] Taniguchi Ishikawa E, Gonzalez-Nieto D, Ghiaur G, Dunn SK, Ficker AM, Murali B (2012). Connexin-43 prevents hematopoietic stem cell senescence through transfer of reactive oxygen species to bone marrow stromal cells. Proc Natl Acad Sci U S A.

[68] Shao Q, Esseltine JL, Huang T, Novielli-Kuntz N, Ching JE, Sampson J (2019). Connexin43 is Dispensable for Early Stage Human Mesenchymal Stem Cell Adipogenic Differentiation But is Protective against Cell Senescence. Biomolecules.

[69] Golan K, Singh AK, Kollet O, Bertagna M, Althoff MJ, Khatib-Massalha E (2020). Bone marrow regeneration requires mitochondrial transfer from donor Cx43-expressing hematopoietic progenitors to stroma. Blood.

[70] Kim JS, Kwon D, Hwang ST, Lee DR, Shim SH, Kim HC (2013). hESC expansion and stemness are independent of connexin forty-three-mediated intercellular communication between hESCs and hASC feeder cells. PloS one.

[71] Tabe Y, Yamamoto S, Saitoh K, Sekihara K, Monma N, Ikeo K (2017). Bone Marrow Adipocytes Facilitate Fatty Acid Oxidation Activating AMPK and a Transcriptional Network Supporting Survival of Acute Monocytic Leukemia Cells. Cancer Res.

[72] Coussens LM, Werb Z (2002). Inflammation and cancer. Nature.

[73] Stevens AJ, Harris AR, Gerdts J, Kim KH, Trentesaux C, Ramirez JT (2023). Programming multicellular assembly with synthetic cell adhesion molecules. Nature.

[74] Guerzoni C, Ferrari-Amorotti G, Bardini M, Mariani SA, Calabretta B (2006). Effects of C/EBPalpha and C/EBPbeta in BCR/ABL-expressing cells: differences and similarities. Cell Cycle (Georgetown, Tex).

[75] Hayashi Y, Hirai H, Kamio N, Yao H, Yoshioka S, Miura Y (2013). C/EBPβ promotes BCR-ABL-mediated myeloid expansion and leukemic stem cell exhaustion. Leukemia.

[76] Kurata M, Onishi I, Takahara T, Yamazaki Y, Ishibashi S, Goitsuka R (2021). C/EBPβ induces B-cell acute lymphoblastic leukemia and cooperates with BLNK mutations. Cancer Sci.

[77] Guerzoni C, Bardini M, Mariani SA, Ferrari-Amorotti G, Neviani P, Panno ML (2006). Inducible activation of CEBPB, a gene negatively regulated by BCR/ABL, inhibits proliferation and promotes differentiation of BCR/ABL-expressing cells. Blood.

[78] Duprez E, Wagner K, Koch H, Tenen DG (2003). C/EBPbeta: a major PML-RARA-responsive gene in retinoic acid-induced differentiation of APL cells. EMBO J.

[79] Machaliński B, Honczarenko M, Gontarewicz A, Ratajczak MZ (1998). [Isolation of human mononuclear cells from bone marrow, peripheral blood and cord blood using Ficoll-Paque (Pharmacia) and Gradisol L (Polfa). Comparative study]. Pol Arch Med Wewn.

[80] Wade MH, Trosko JE, Schindler M (1986). A fluorescence photobleaching assay of gap junction-mediated communication between human cells. Science (New York, NY).

